# The Inorganic Side of NGF: Copper(II) and Zinc(II) Affect the NGF Mimicking Signaling of the N-Terminus Peptides Encompassing the Recognition Domain of TrkA Receptor

**DOI:** 10.3389/fnins.2016.00569

**Published:** 2016-12-20

**Authors:** Giuseppe Pandini, Cristina Satriano, Adriana Pietropaolo, Fiorenza Gianì, Alessio Travaglia, Diego La Mendola, Vincenzo G. Nicoletti, Enrico Rizzarelli

**Affiliations:** ^1^Endocrinology, Department of Clinical and Experimental Medicine, Garibaldi-Nesima Medical Center, University of CataniaCatania, Italy; ^2^Institute of Biostructures and Bioimages - Catania, National Research CouncilCatania, Italy; ^3^Department of Chemical Sciences, University of CataniaCatania, Italy; ^4^Consorzio Interuniversitario di Ricerca in Chimica dei Metalli nei Sistemi BiologiciBari, Italy; ^5^Department of Health Sciences, University of CatanzaroCatanzaro, Italy; ^6^Centre for Neural Science, New York UniversityNew York, NY, USA; ^7^Department of Pharmacy, University of PisaPisa, Italy; ^8^Section of Medical Biochemistry, Department of Biomedical and Biotechnological Sciences (BIOMETEC), University of CataniaCatania, Italy

**Keywords:** neurotrophins, metal ions, ionophore, CREB, BDNF, peptidomimetics, Alzheimer's disease, nanomedicine

## Abstract

The nerve growth factor (NGF) N-terminus peptide, NGF(1–14), and its acetylated form, Ac-NGF(1–14), were investigated to scrutinize the ability of this neurotrophin domain to mimic the whole protein. Theoretical calculations demonstrated that non-covalent forces assist the molecular recognition of TrkA receptor by both peptides. Combined parallel tempering/docking simulations discriminated the effect of the N-terminal acetylation on the recognition of NGF(1–14) by the domain 5 of TrkA (TrkA-D5). Experimental findings demonstrated that both NGF(1–14) and Ac-NGF(1–14) activate TrkA signaling pathways essential for neuronal survival. The NGF-induced TrkA internalization was slightly inhibited in the presence of Cu^2+^ and Zn^2+^ ions, whereas the metal ions elicited the NGF(1–14)-induced internalization of TrkA and no significant differences were found in the weak Ac-NGF(1–14)-induced receptor internalization. The crucial role of the metals was confirmed by experiments with the metal-chelator bathocuproine disulfonic acid, which showed different inhibitory effects in the signaling cascade, due to different metal affinity of NGF, NGF(1–14) and Ac-NGF(1–14). The NGF signaling cascade, activated by the two peptides, induced CREB phosphorylation, but the copper addition further stimulated the Akt, ERK and CREB phosphorylation in the presence of NGF and NGF(1–14) only. A dynamic and quick influx of both peptides into PC12 cells was tracked by live cell imaging with confocal microscopy. A significant role of copper ions was found in the modulation of peptide sub-cellular localization, especially at the nuclear level. Furthermore, a strong copper ionophoric ability of NGF(1–14) was measured. The Ac-NGF(1–14) peptide, which binds copper ions with a lower stability constant than NGF(1–14), exhibited a lower nuclear localization with respect to the total cellular uptake. These findings were correlated to the metal-induced increase of CREB and BDNF expression caused by NGF(1–14) stimulation. In summary, we here validated NGF(1–14) and Ac-NGF(1–14) as first examples of monomer and linear peptides able to activate the NGF-TrkA signaling cascade. Metal ions modulated the activity of both NGF protein and the NGF-mimicking peptides. Such findings demonstrated that NGF(1–14) sequence can reproduce the signal transduction of whole protein, therefore representing a very promising drug candidate for further pre-clinical studies.

## Introduction

Neurotrophins are a family of structurally conserved growth factors involved in differentiation, survival of neurons (Huang and Reichardt, 2001; Chao, 2003) as well as of non-neuronal cell type (Sofroniew et al., 2001; Reichardt, 2006; Caporali and Emanueli, 2009). They include nerve growth factor (NGF), brain derived neurotrophic factor (BDNF), NT-3 (neurotrophin 3), and NT-4.

Neurotrophins exert their biological functions mainly via two types of cell membrane receptors: the Trk (tyrosine receptors kinase) and the common neurotrophin receptor P75NTR.

NGF activates TrkA receptor triggering downstream signaling pathways (Kaplan and Miller, 2000; Chao, 2003; Huang and Reichardt, 2003), while p75NTR signaling is complex (Skeldal et al., 2011), inducing both survival and apoptosis mechanisms (Salehi et al., 2000; Roux et al., 2001; Mamidipudi et al., 2002).

NGF, composed of 118 amino acid residues (Angeletti et al., 1971) has been discovered in the 1950s (Levi-Montalcini and Hamburger, 1951; Levi-Montalcini, 1952; Cohen et al., 1954; Levi-Montalcini and Booker, 1960); it displays multiple physiological actions in the central nervous system, showing neurotrophic effects and resulting critical for the neurite outgrowth and survival and maintenance of neurons (Hu et al., 2005; Tucker et al., 2008; Xu et al., 2012).

Most importantly, NGF has strong anti-apoptotic effects and, under its deprivation, neurons exhibit a series of morphological changes and eventually undergo apoptosis (Lomb et al., 2009).

The clinical significance of NGF has been extensively investigated. It is well known that NGF profoundly affects the development of both young and adult nervous systems (Tuszynski and Blesch, 2004). In the central nervous system (CNS), NGF dysregulation has been correlated to several neuronal degeneration diseases, including Alzheimer's disease and multiple sclerosis (Biernacki et al., 2005; Cattaneo and Calissano, 2012). Besides its role in the CNS, there is evidence that NGF acts throughout the body and plays roles in many organs (Levi-Montalcini, 2004) and related disorders (Chaldakov et al., 2001, 2004; Manni et al., 2005; Cheng et al., 2012).

Therefore, the delivery of NGF to the target region might rescue these pathologies or alleviate the symptoms. However, the pleiotropic actions of neurotrophins, due to the activation of their multireceptor signaling networks, are also responsible of adverse effects, such as activation of p75 pathway (Fahnestock et al., 2001; Mufson et al., 2012) or pain (Dyck et al., 1997; Bergmann et al., 1998) with further issues for their clinical applications. In addition, NGF has low bioactive stability in the body, possesses limited blood–brain barrier (BBB) permeability, thus limiting its use as a neuroprotective drug (Akassoglou, 2005; Weissmiller and Wu, 2012).

To overcome these limitations, alternative strategies involve either *ex vivo* gene delivery or biologically stable small molecules that could bind and activate TrkA signaling pathway (Massa et al., 2003; Tuszynski et al., 2005).

The characterization of the structure of TrkA receptor (Ultsch et al., 1999), as well as the structure of NGF bound to the TrkA Ig-domain (Wiesmann et al., 1999) allowed for the identification of the residues that account for the specificity observed in the NGF-TrkA interaction (Urfer et al., 1998; Wiesmann and de Vos, 2001). Such finding favored the design and the development of small-molecule (Chen et al., 2001) that could exert: (i) therapeutic beneficial effects on neuronal and synaptic plasticity; (ii) suitable pharmacokinetics and CNS penetration for drug development, without unwanted systemic effects produced by the full-length protein (Xie and Longo, 2000; Massa et al., 2002, 2003; Longo and Massa, 2004, 2005, 2013).

First attempts to develop small-molecule mimetics of neurotrophic factors have been focused on the synthesis of small peptides encompassing amino acids residues of various NGF domains (Longo and Massa, 2013). The first small peptide molecule corresponding to an NGF domain, that demonstrated to exert a neurotrophic effect, has been a cyclic dimeric mimic peptide (amino acid residues, KGKE) able to interact with the p75NTR receptor (Longo et al., 1997). NGF small mimic peptide containing KGKE or a homologous sequence blocked Aβ binding to p75NTR and protected against Aβ-induced cell death (Yaar et al., 2007). Another NGF small peptide fragment, which encompasses the amino acids of NGF β-turn loops and acts through TrkA receptor, rescued basal forebrain cholinergic neurodegeneration, spatial reference memory (Bruno et al., 2004) and short-term memory deficits (Aboulkassim et al., 2011). Among the NGF different domains, the N-terminus tail resulted to play a crucial role for TrkA receptor binding and activation (Kahle et al., 1992; Shih et al., 1994). In particular, biological and computational findings identified His-4, His-8, Ile-6, Phe-7, and Glu-11 as critical residues for this interaction (Woo and Neet, 1996; Berrera et al., 2006).

**Scheme 1:**
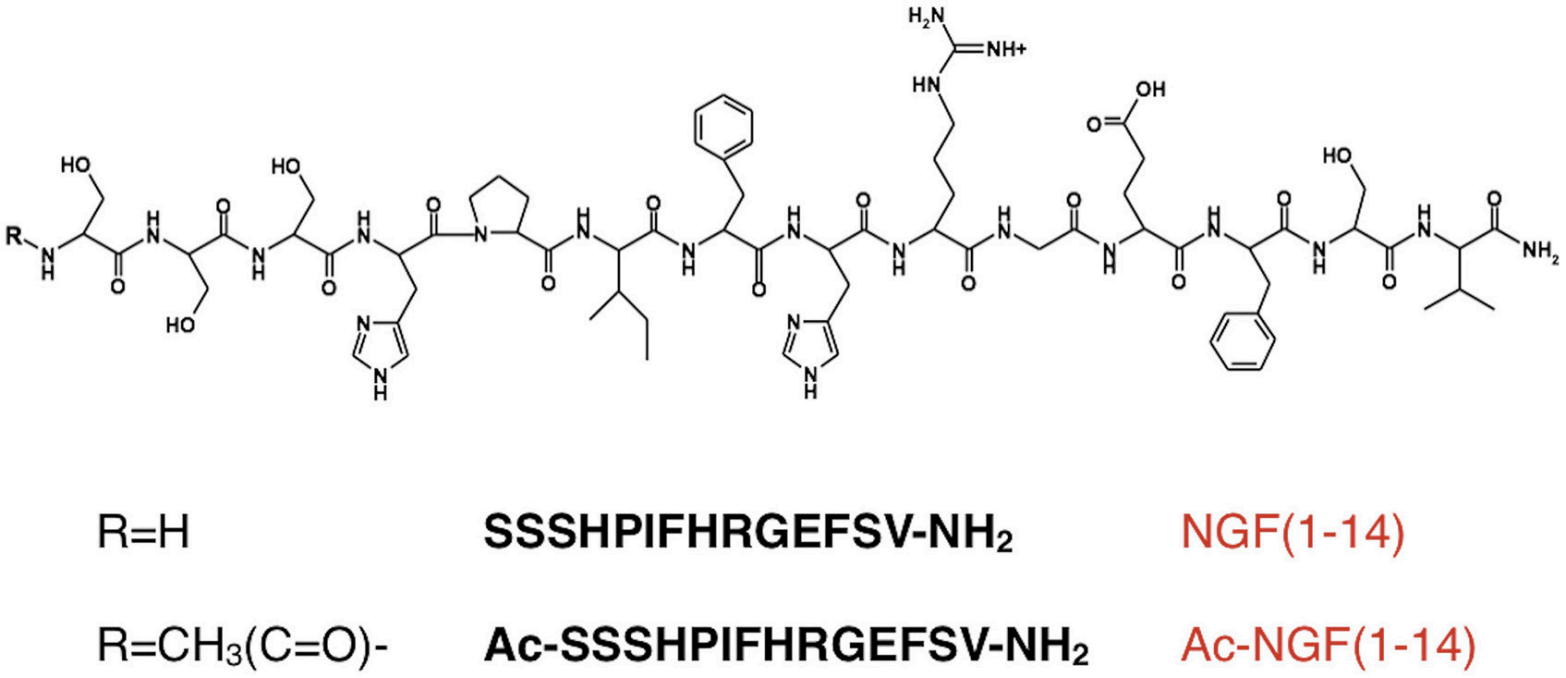
**Schematic representation of NGF(1–14) and Ac-NGF(1–14) peptides**.

Recently, a small peptide encompassing the 1–14 sequence of the human NGF (NGF(1–14)) (Scheme 1; Travaglia et al., 2013b, 2015), has been shown to activate TrkA receptor, partly inducing its downstream signaling cascade in PC12 cells. The peptide affected the phosphorylation of PI3-K, Akt, GSK-3 (Yao and Cooper, 1995; Cantley, 2002), with effects largely comparable with those induced by NGF. In addition, NGF(1–14) triggered the phosphorylation of the transcription factor cAMP response element-binding protein (CREB), which represents a major transcriptional mediator of neuronal responses to neurotrophins (Finkbeiner et al., 1997), axonal regeneration (Teng and Tang, 2006), memory consolidation (Alberini, 2009; Kim et al., 2013; Bisaz et al., 2014) as well as metabolism (Leone et al., 2011).

A large body of literature indicates that block d metal ions not only affect the pathways involved in the expression of transcription factors as CREB (Newton et al., 2000; Kalkhoven et al., 2002; Liu et al., 2008) but also influence the NGF as well as other neurotrophins' signaling (Travaglia et al., 2011). Zinc and copper ions modulate the level of NGF (Hwang et al., 2007) that decreases in zinc(II) deficient mice (Kheirvari et al., 2008), while zinc(II) dietary supplementation seems to replenish the NGF level with unknown mechanisms (Kheirvari et al., 2006).

Zn^2+^ and Cu^2+^ high concentrations inhibit the *in vitro* effects of NGF (as well as BDNF, NT-3, and NT-4/5), inducing conformational changes that alter the NGF binding to its receptor TrkA. NGF metal ion binding has been shown: (i) to block the NGF-mediated neurite outgrowth in chick dorsal root ganglia (DRG); (ii) to decrease the cell viability (Maitra et al., 2000); and (iii) to counteract the NGF-mediated protection from oxidative stress in pheochromocytoma (PC12) cells (Ross et al., 1997; Wang, 1999). Conversely, Zn^2+^ acts as a key factor for the protective activity of NGF (Zhao et al., 2004), showing that metal loading on native NGF increases its ability to trigger TF1 cell proliferation and mediates PC12 cell survival. Zn^2+^ and Cu^2+^ antagonize p75-driven apoptosis in chick neural retina (Allington et al., 2001) and block the NGF binding to p75, attenuating its pro-apoptotic signaling cascade in chick embryonic cell cultures.

NGF is able to increase the cellular level of copper in PC12 cells within 3 days of treatment up to 14-fold (Birkaya and Aletta, 2005) and copper complexes with ionophore ligands promote neurite elongation with neuroprotective and neurogenerative processes, related to the intracellular delivery or redistribution of the biometal (Bica et al., 2014).

The Zn^2+^ and Cu^2+^ complexes with peptide fragments encompassing both the NGF N-terminal sequence 1–14 and its N-acetylated derivative Ac-NGF(1–14) (Scheme 1) have been characterized. It has been shown that the two metal ions bind the N-terminal domain with different coordination modes (Travaglia et al., 2011).

Furthermore, Ac-NGF(1–14) shows metal complex stability constant values lower than those of NGF(1–14), up to two order of magnitude lower for Cu^2+^ complex species.

The difference in coordination feature might explain the biological effect of these peptides in presence of metal ions. In fact, both peptides displayed a proliferative effect on SHSY5Y cells, in a similar way to that observed for the NGF protein. However, the proliferative effect of NGF(1–14) significantly increased in the presence of Zn^2+^ or Cu^2+^, whereas the activity of Ac-NGF(1–14) resulted practically unaffected under the same experimental conditions. Furthermore, while SHSY5Y cell culture treated with NGF and copper showed a synergic increase in the cell number, on the contrary, co-treatment with zinc and murine NGF inhibited the cell growth (Travaglia et al., 2011, 2012b).

Here we report on: (i) the ability of Ac-NGF(1–14) to activate TrkA signaling on PC12 cells, together a re-evaluation of NGF(1–14) behavior; (ii) the ionophore capacity of the two peptides; (iii) the role played by Cu^2+^ and Zn^2+^ on NGF and related mimicking peptides. The Ac-NGF(1–14) binding mode to the TrkA receptor was also determined by means of a computational study.

## Materials and methods

### Peptide synthesis

The peptide encompassing the amino acid sequences SSSHPIFHRGESFV-NH_2_, NGF(1- 14), and its acetylated form, Ac-SSSHPIFHRGESFV-NH_2_, Ac-NGF(1–14), were synthesized with the C-termini amidated as previously reported (Travaglia et al., 2011). The fluorescent peptides, NGF(1–14)FAM and Ac-NGF(1–14)FAM, were labeled with 5,6-carboxyfluorescein through the side chain of an amidated additional lysine residue (FAM) (purchased from CASLO, Lyngby, Denmark).

### Theoretical calculations

#### Parallel tempering simulations

Ac-NGF(1–14) underwent 20 ns of parallel tempering (PT) simulations in explicit solvent with a total volume of 40 × 40 × 40 Å^3^, after the equilibration through 2 ns of MD in explicit solvent. GROMACS 4.5.6 package (Hess, 2008) was used. The overall charge of the system was neutralized by adding 1 chloride ion. Periodic boundary conditions were applied. The AMBER99SB (Hornak et al., 2006) force field was used for the acetylated peptide and counter ions, and the TIP3P (Jorgensen et al., 1983) force field was used for water molecules. Electrostatic interactions were calculated using the Particle Mesh Ewald method (Essmann et al., 1995). A cutoff (0.9 nm) was used for the Lennard-Jones interactions. The time-step was set to 2 fs. All bond lengths were constrained to their equilibrium values using the SHAKE (Miyamoto and Kollman, 1992) algorithm for water and the LINCS (Hess, 2008) algorithm for the peptide. We simulated 64 replicas distributed in the temperature range 300–400 K following a geometric progression. All replicas were simulated in NVT ensemble using a stochastic thermostat (Bussi et al., 2007) with a coupling time of 0.1 ps. A thermostat that yields the correct energy fluctuations of the canonical ensemble is crucial in parallel tempering simulations (Rosta et al., 2009). Exchanges were attempted every 0.1 ps. The method of Daura and Van Gunsteren (Daura et al., 1999) was used in post-processing phase to cluster the resulting trajectories, with a cutoff of 3 Å calculated on the backbone atoms as implemented in the clustering utility provided in the GROMACS package (Hess, 2008). This simulation protocol has successfully been tested in predicting the conformational features of small peptides (Travaglia et al., 2012a, 2013a; Pietropaolo et al., 2015), combined with unbiased simulations for disclosing the structural packing enhanced by specific residues (Pietropaolo et al., 2007, 2008).

#### Docking simulations

The starting coordinates of domain-5 of TrkA (TrkA-D5) were taken from the Xray structure of TrkA-D5 bound to NGF (pdb code 1WWW) (Wiesmann et al., 1999). The former complex was used as template for the alignment of the main MD clusters of Ac-NGF(1–14) prior to the docking to TrkA-D5. Docking simulations were performed using HADDOCK interface (de Vries et al., 2010). All residues of Ac-NGF(1–14) were included as active residues for the Haddock docking, as well as V288 to C300 belonging to TrkA-D5. Structures underwent rigid body energy minimization, semirigid simulated annealing in torsion angle space, with a final clusterization of the results. This docking protocol has successfully been tested in predicting the binding modes concerning protein/peptide interactions (Bellia et al., 2013; Grasso et al., 2015).

### Biological assays

#### Cell cultures

Cell media and chemicals, unless otherwise stated, were obtained from Sigma (St. Louis, MO). Fetal bovin serum (FBS), horse serum (HS), and NGF were obtained from Invitrogen Laboratories (Paisley, U.K.). NGF was used at a concentration of 50 ng/ml (approximately 4 × 10^−9^ M). Anti-phospho-TrkA (Y490) (Cat # 9141), anti-TrkA (Cat # 2505), anti-phospho-ERK1/2 (T202/Y204) (Cat # 9106), anti-ERK (Cat # 9107), anti-phospho-AKT (S473) (Cat # 4051), anti-AKT (Cat # 4685), anti-phospho-CREB (S133) (Cat # 9191), anti-CREB (Cat # 9197), and anti-P75^NTR^ (Cat # 4201) antibodies were from Cell Signaling Technology (Danvers, MA). Anti-Grb2 antibody (Cat # sc-17813) was purchased from Santa Cruz Biotechnology (Santa Cruz, CA). Rat pheochromocytoma (PC12) cells were obtained from the American Type Culture Collection (Manassas, VA), and cultured, at passages between the 5 and 20, in RPMI-1640 (GIBCO), supplemented with 10% horse serum (HS), 5% fetal bovine serum (FBS), 2 mM L-glutamine, 50 IU/ml penicillin, and 50 μg/ml streptomycin.

#### Real-time PCR

Total RNA (5 μg) from PC12 cells, isolated using RNeasy Mini Kit (Qiagen; Hilden, Germany) in accordance to the manufacturer instructions and treated with RNase-free DNase I (Qiagen; Hilden, Germany), was reverse transcribed with ThermoScript RT (Invitrogen) and Oligo dT primers. Synthesized cDNA (25 ng) was then combined in a PCR reaction using the appropriate primers and probes. Quantitative real-time PCR for the expression of neuronal specific differentiation markers was performed using TaqMan® Gene expression Assays: Gap43(Rn01474579_m1), Elavl4(Rn01416883_m1), Map2(Rn00565046_m1), Tubb3(Rn01431594_m1), B2m(Rn00560865_m1), according to the manufacturer's instructions. Quantitative real-time PCR for the expression of BDNF was performed using SYBR Green PCR Master Mix (PE Applied Biosystems) with the following primers: BDNF-Forward 5′-TCA AGC TGG AAG CCT GAA TGA A-3′, BDNF-reverse 5′-CCC AGT CAG GTA ACC ACT AAC AC-3′, using B2m as gene housekeeping: B2m-forward 5′-CCC ACC CTC ATG GCT ACT TC-3′, B2m-reverse 5′-GAT GAA AAC CGC ACA CAG GC-3′. Amplification reactions was performed on an ABI Prism 7500 (PE Applied Biosystems) according to the manufacturer's instructions. Relative quantitative determination of target gene levels was done by comparing ΔCt.

#### Western blot analysis

PC12 cells un-pre-treated or pre-treated 30 min before with CuSO_4_ or ZnSO_4_ 1μM were stimulated with NGF 50 ng/ml, NGF(1–14) or Ac-NGF(1–14) 50 μM for 5, 15, and 30 min. After treatments, cells were washed with ice cold PBS and lysed with cold RIPA buffer (50 mM Tris pH 7.4, 150 mM NaCl, 1% Triton X-100, 0.25% sodium deoxycolate, 10 mM sodium pyrophosphate, 1 mM NaF, 1 mM sodium orthovanadate, 2 mM PMSF) in the presence of phosphatase and protease inhibitor cocktails (Roche), and the insoluble material separated by centrifugation at 10,000 × g for 15 min at 4°C.

Cell lysates were subjected to SDS-PAGE and the resolved proteins were transferred to nitrocellulose membranes, immunoblotted with phospho-specific antibodies and detected by ECL. The nitrocellulose membrane was then stripped with buffer Restore® (Pierce, Rockford, IL) and, subsequently, reprobed with the specific antibodies for the unphosphorylated proteins and the Grab2 antibody to control for protein loading. Quantitative densitometric analysis was performed using ImageJ (US National Institutes of Health). Grb2 was used as loading control for all markers. The level of phosphorylation was calculated as ratio between data from anti-phospho antibodies over those from the related not phosphorylated counterparts.

To assay internalization of TrkA and P75^NTR^ receptors, PC12 cells, both untreated and pre-treated 30 min before with 1 μM of CuSO_4_ or ZnSO_4_ were stimulated for 30 min with 50 ng/mL protein (i.e., 4 nM NGF) or 10 μM peptides (NGF(1–14) or Ac-NGF(1–14)) in DMEM with 5 mM HEPES and 0.1% BSA. Cells were rinsed with cold PBS and incubated with 1.5 mg/ml EZ-Link Sulfo-NHS-LC-Biotin [sulfosuccinimidyl-6-(biotinamido)hexanoate] (Pierce, Rockford, IL) in biotinylation buffer (10 mM boric acid, 154 mM NaCl, 7.2 mM KCl, 1.8 mM CaCl_2_, pH 8.4) for 30 min. Cells were then rinsed twice with quenching buffer (192 mM glycine, 25 mM Tris-HCl, 1.8 mM CaCl_2_, 154 mM NaCl, pH 8.3) and lysed in lysis buffer (20 mM Tris-HCl, pH 7.5, 137 mM NaCl, 1% Nonidet P-40, 10% glycerol, 1 mM MgCl_2_, 1 mM EGTA, 1 mM Na3VO4, 20 mM_-glycerol phosphate, 20 mM NaF, 1 mM PMSF, and 1 mg/ml aprotinin and leupeptin). Lysates were incubated on ice for 10 min and then centrifuged at 13,000 × g for 10 min at 4°C. Protein concentrations were determined by the Bradford method. Lysates containing 1 mg of protein each were added to 100 μl of UltraLink Immobilized NeutrAvidin beads (Pierce, Rockford, IL). Samples were incubated with rocking at 4°C for 2 h. NeutrAvidin beads were rinsed three times with lysis buffer, and 50 μl of Laemmli buffer was added to each sample. Samples were resolved by SDSPAGE and immunoblotted for TrkA and P75^NTR^.

#### Statistical analysis

Statistical analysis was performed with one-way ANOVA, followed by the Bonferroni test. Data are expressed as mean ± SEM. Statistical significance was accepted at the 95% confidence level (*P* < 0.05).

### Laser scanning confocal microscopy (LSM)

A FV1000 laser-scanning microscope (Olympus), equipped with diode (LD405), Argon multiline (458, 488, 515) and HeNe (543 and 633) lasers, fitted with a low chromatic aberration objective PlanApo60x (NA: 1.4, oil immersion, W.D.: 0.12 mm) was used. The images were scanned with Kalman filtering on at the resolution of 512 × 512 pixels. For multichannel imaging, fluorescent dyes were imaged sequentially to eliminate cross talk between the channels, namely: (i) the blue (ex405/em 425–475), for the emission of the DAPI-stained nuclei, (ii) the green (ex488/em 500–530), for the emission of the carboxyfluorescein (FAM) group in the NGF(1–14)FAM and Ac-NGF(1–14)FAM peptides, and (iii) the red (ex543/em 560–700), for the BODIPY moiety of CS1 copper probe (Miller et al., 2006).

PC12 cells were seeded on glass bottom dishes (WillCo Wells B.V., Amsterdam, NL), pre-coated with 0.01% polylysine, at a density of 5 × 10^4^, and maintained in D-MEM complete medium for 24–36 h. The day of the experiment cells were rinsed with serum-free medium.

Live cell imaging experiments of the peptide cellular uptake were performed directly on the microscope stage operating in xy-time scan mode, with a total scan time of 20 min and a scan interval set to 30 s. More in detail, after the first 1–2 scans, peptides were quickly added from a 100X concentrated stock solution in water to the Petri dish containing the cells in 1 mL DMEM. The solution was vigorously mixed to ensure a homogeneous peptide dilution to the final concentration of 10 μM in the whole liquid volume, in order to avoid artifacts in the real time live cell imaging owing to different diffusion rates of the peptide molecules related to concentration gradients. The acquisition parameters were kept constant for all the experiments. Confocal imaging of metal trafficking was performed by treatments of PC12 cell for 5 min with the peptide solution (10 μM), either NGF(1–14) or Ac-NGF(1–14) in DMEM. After that, cells were rinsed (2 × 1mL) with phosphate buffer saline solution (PBS, 10 mM, pH = 7.4 at 25°C) and stained by 15 min incubation with CS1 intracellular monovalent copper probe (final concentration of 2 μM from 1 mM stock solution in DMSO) and the cell-permeant nuclear counterstain Hoechst 33342 (NucBlue® Live ReadyProbes® Reagent, Life Technologies), followed by buffer rinsing (2 × 1 mL). In two parallel experiments, before the treatments with the peptide NGF(1–14) and Ac-NGF(1–14), cells were pre-incubated in the medium supplemented with 50 μM of copper chelator BCS (bathocuproine disulfonic acid disodium salt, purchased from Sigma Aldrich, from a 10 mM stock solution in DMSO) for 3 h or 100 μM of CuSO_4_ for 1 h. A 0.5% (v/v) DMSO was added in all conditions as control. At the end of treatments and staining, the cells were fixed in fresh 2% paraformaldehyde and deeply rinsed (3 × 2 mL) with PBS.

#### Confocal microscopy images analysis

The nuclear and whole cell areas were bordered with regions of interest (ROIs). About five separate scans were performed on every dish, and each scan comprised an average of 10 cells. Thus, at least 50 cells were sampled per data point and each experiment was repeated at least three times.

Quantitative analysis of fluorescence was performed by using the ImageJ software (1.50i version, NIH), in terms of integrated density ID = *N*·[*M* − *B*], where *N* is the number of pixels in the selection, *M* is the average gray value of the pixels and *B* is the most common pixel value (Satriano et al., 2003). The values obtained by these analyses were imported into OriginPro 8.6 program for statistical analysis for *P*-values, calculated by using a one-way ANOVA with a Tukey multiple comparison test.

## Results

### Conformational ensemble of Ac-NGF(1–14) and its interaction with TrkA-D5 shows a larger number of non-covalent interactions than for NGF(1–14)

The N-terminal domain of NGF binds specifically to domain 5 of TrkA (TrkA-D5), affording distinct weak non-covalent interactions (Travaglia et al., 2015). Here we aimed to assess, through molecular simulations, whether the protection of the N-terminal amino group by acetylation alters the structural features of NGF(1–14) upon the binding toTrKA-D5.

Analogously with the simulations carried out for the wild-type NGF(1–14), parallel tempering simulations were run in parallel, in order to sample the conformations of Ac-NGF(1–14) at physiological pH. Three main clusters were observed accounting for 68%, 22%, and 10% of population percentage.

**Figure 1:**
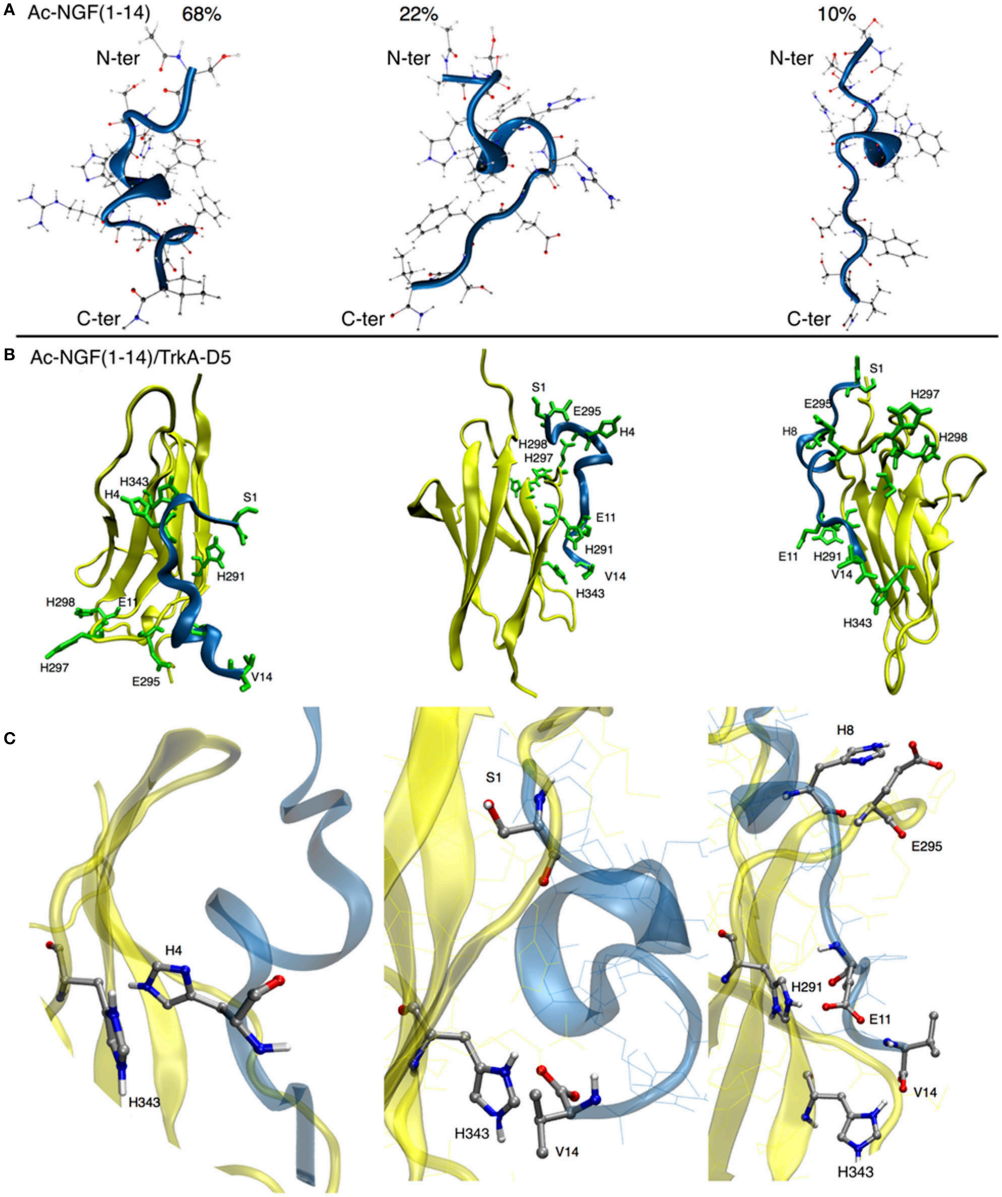
**(A)** The three main clusters of Ac-NGF(1–14). Carbon atoms are shown in gray, nitrogen atoms in blue and oxygen atoms in red spheres. Ac-NGF(1–14) is shown in blue and TrkA-D5 is shown in yellow. N- and C-termini are shown from up to bottom, respectively. The population percentage of each cluster is shown. **(B)** The three lowest energy binding modes for the Ac-NGF(1–14)/TrkA-D5. Ac-NGF(1–14) domains are shown by blue ribbons, TrkA-D5 regions are shown by yellow ribbons. Ac-NGF(1–14) and TrkA-D5 backbone are shown by sticks. **(C)** The relevant contact interactions between Ac-NGF(1–14) and TrkA-D5.

The simulated conformations showed a tendency to preserve a loop state that can be further stabilized via backbone hydrogen bonds in the central domain, to form a short alpha helix (Figure 1A). Such an alpha helix conformation, also observed in NGF(1–14) (Travaglia et al., 2015), was thus preserved upon the acetylation of the N-terminal amino group. In general, the various conformational states of Ac-NGF(1–14) were not affected from the N-terminal acetylation. However, upon docking the Ac-NGF(1–14) with TrkA-D5 relevant differences were observed in the three main binding poses (Figure 1B).

In the first binding pose, the acetylation of the amine group caused S1 moving far away from TrkA-D5, resulting in a different rearrangement of the residues of Ac-NGF(1–14); H4 faces H343 of TrkA-D5, at variance with the contact with H291 observed in NGF(1–14).

The second binding pose indicated that V14 of Ac-NGF(1–14) approaches H343 of TrkA-D5, a contact also observed in NGF(1–14). However, the contacts involving S1 of NGF(1–14) with TrkA-D5 were completely absent in Ac-NGF(1–14), owing to the acetylation of the N-terminal amine group.

The third binding pose highlighted a close contact of H8 of Ac-NGF(1–14) with E295 of TrkA-D5, E11 of Ac-NGF(1–14) with H291 of TrKA-D5 and a C-terminal contact involving V14 of AcNGF(1–14) with H343 of TrkA-D5. These contacts, observed also in NGF(1–14), were consistent with the other binding poses and, at variance with NGF(1–14), the S1 residue of Ac-NGF(1–14) was not facing any residues of TrkA-D5 (Figure 1C).

**Figure 2:**
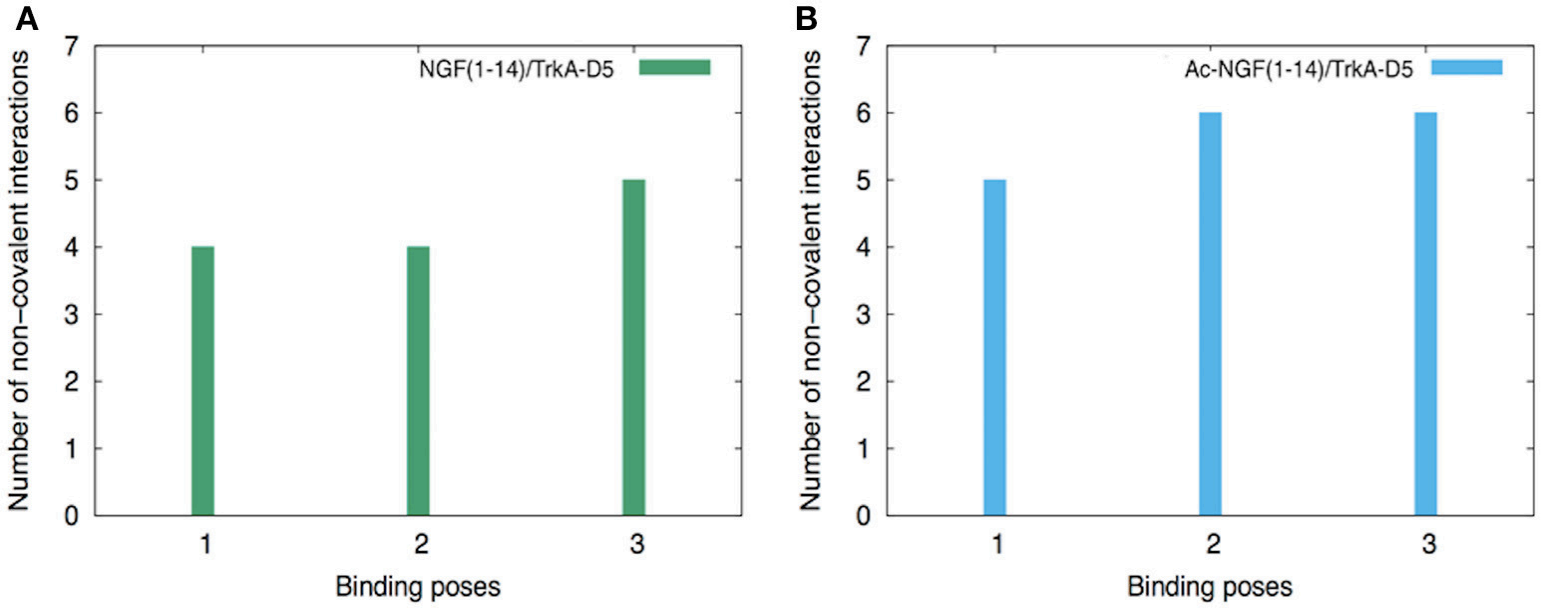
**The histograms of the number of non-covalent interactions calculated for NGF(1–14) (A)** and Ac-NGF(1–14) **(B)** bound to TrkA-D5.

It is worth noting that the protection of the free-amino N-terminal group leads to the adoption of hydrogen bonds and salt-bridge interactions between the Ac-NGF(1–14) peptide and TrkA-D5. The number of non-covalent interactions was slightly higher for the Ac-NGF(1–14), if compared with those formed in NGF(1–14) peptide (Figure 2). Specifically, the following interactions were involved: (i) the OH groups of S1 and S2 side chains with the carbonyl groups of A293, E295 of TrkA-D5, (ii) the H4 imidazole hydrogen with the OH group of S304 of TrkA-D5, (iii) the E11 carboxyl group both with R289 amine side chain residue, forming salt-bridge. The E11 carboxyl group can also be engaged in a hydrogen bond with the imidazole hydrogen of H291 of TrkA-D5.

Interestingly, both peptides show the tendency to form a higher number of non-covalent interactions than in the co-crystallized protein structure (Wiesmann et al., 1999).

The acetylation of the N-terminal amino group of NGF(1–14) thus induces distinct structural features toward the binding with TrkA-D5, with a tendency to form weak non-covalent interactions.

### Effect of NGF(1–14) and Ac-NGF(1–14) on morphology and differentiation, in the presence or absence of CuSO_4_ and ZnSO_4_

**Figure 3:**
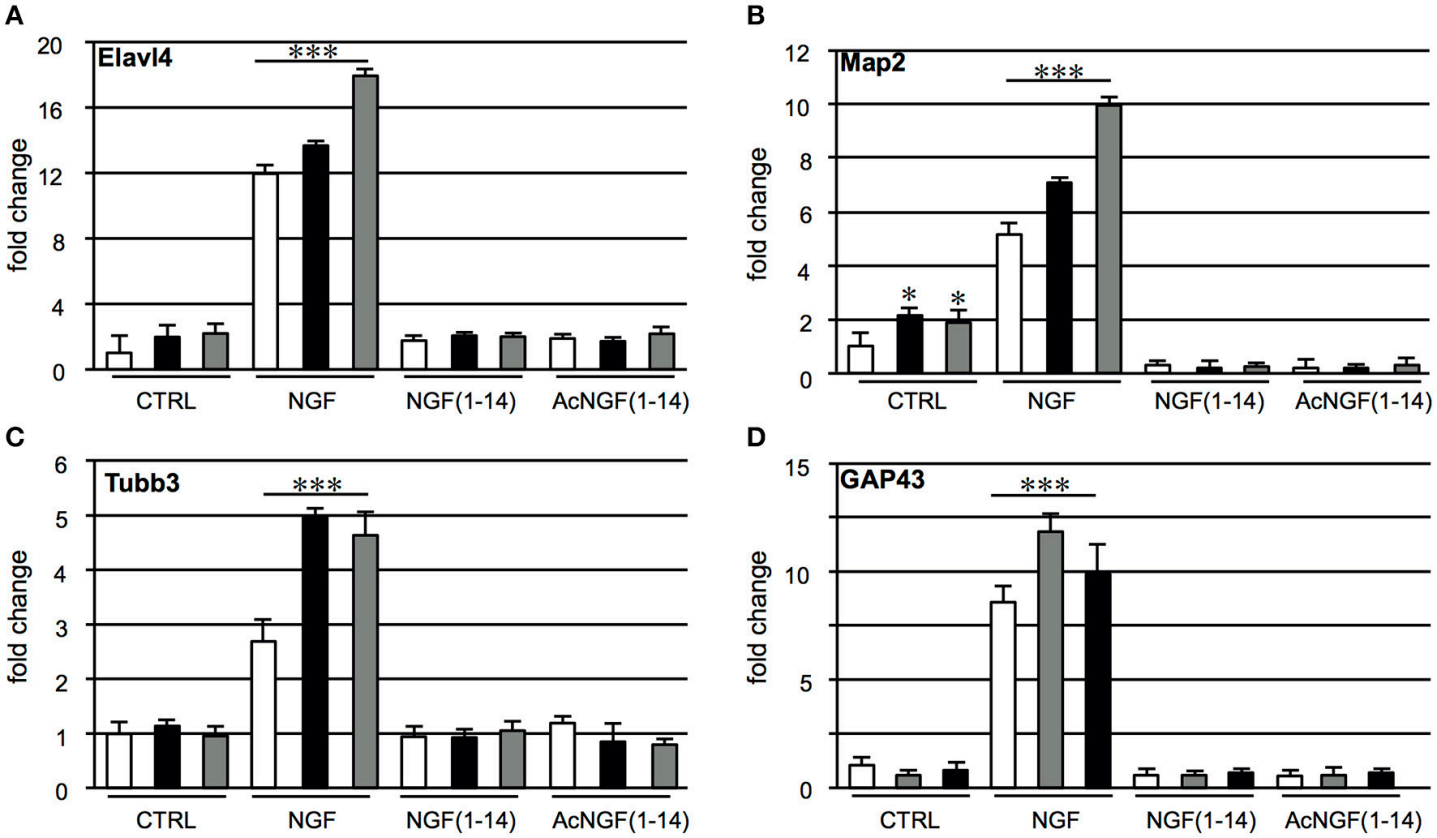
**Effect of NGF(1–14) on the expression of specific neuronal differentiation markers in PC12 cells**. Levels of: **(A)** Elavl4, **(B)** Map2, **(C)** Tubb3, and **(D)** GAP43 mRNA determinated by real-time quantitative RT-PCR. PC12 cells incubated for 3 days with NGF, NGF(1–14) and AcNGF(1–14) in absence (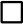) or in the presence of CuSO_4_ (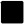) and ZnSO_4_ (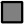). Data normalized with respect to the expression level of B2M mRNA. Results are given as fold-chang of untreated cells. Data are the mean ± SEM of four independent experiments (^*^P < 0.05 w.r.t. control; ^***^P < 0.001 w.r.t. control).

In PC12 cells neurite outgrowth was used as a marker of differentiation. We recently showed that NGF(1–14) was unable to induce a visible differentiated phenotype (Travaglia et al., 2015). Since the neurite outgrowth is a macro marker of neuronal differentiation, the profile expression of specific genes involved in neuronal develop and differentiation, Map2, Tubb3, Elavl4, GAP-43 were evaluated in PC12 cells, untreated or treated for 3 days with the protein (NGF, 50 ng/ml) or the peptides (NGF(1–14) or Ac-NGF(1–14), 10 μM) in the presence or absence of CuSO_4_ or ZnSO_4_ (1 μM) (Figure 3). CuSO_4_ or ZnSO_4_ induced a two-fold increase of the Map2 and Elavl4 mRNA levels, although only for Map2 were significant (Figures 3A,B), while NGF elicited a 2.5-12-fold increase of Map2, Tubb3, Elavl4, and GAP-43 mRNA levels compared to the untreated control (*p* < 0.001). This effect significantly enhanced, up to two-fold, by CuSO_4_ or ZnSO_4_ addition. Conversely, Map2, Tubb3, Elavl4, and GAP-43 mRNA levels were not affected by CuSO_4_ and ZnSO_4_ addition in the cells stimulated with NGF(1–14) or Ac-NGF(1–14).

### NGF(1–14) induces TrkA receptor internalization in PC12 cells only in the presence of CuSO_4_ and ZnSO_4_

Endocytic trafficking of neurotrophins and their receptors is a fundamental feature of neurotrophin signaling. It is unclear whether the different receptor localization causes different intracellular pathway and thus different biological activities. Literature data show that neuronal differentiation requires the internalization of the ligand-receptor complex to clathrin-coated vesicles, while cell survival is induced by receptors located on the cell membrane.

**Figure 4:**
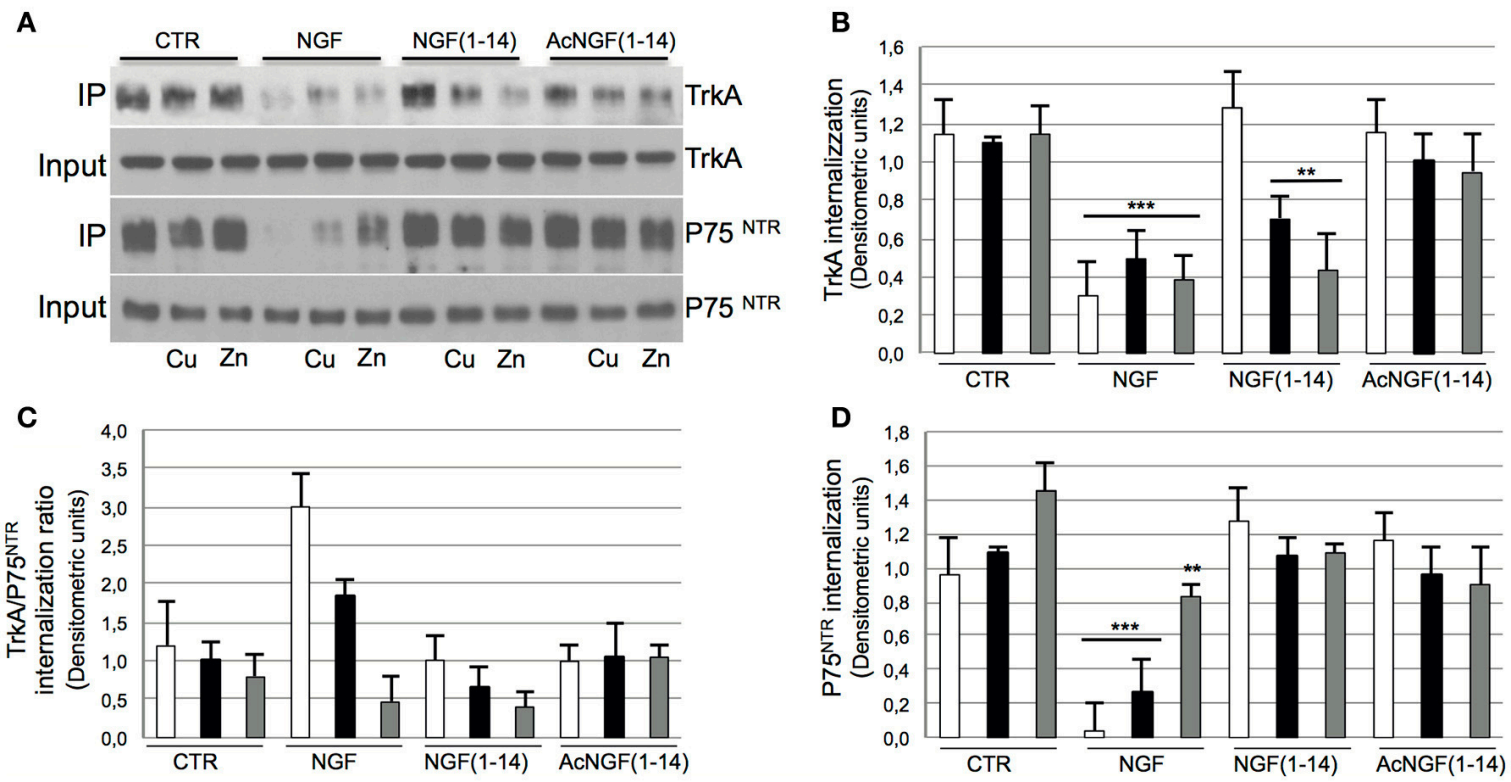
**Effect of NGF(1–14) on internalization of TrkA receptor of PC12 cells in the presence or absence of CuSO_**4**_ and ZnSO_**4**_**. PC12 cells un-pre-treated (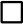) or pre-treated 30 min before with 1 μM CuSO_4_ (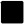) or ZnSO_4_ (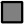) and stimulated with 50 ng/ml NGF, 10 μM NGF(1–14) or 10 μM AcNGF(1–14) for 30 min. **(A)** Western blotting, with blots probed for TrkA and P75^NTR^. Crude cellular lysates run in parallel for input level of TrkA and P75^NTR^. Densitometric analysis of: **(B)** TrkA receptor, **(C)** P75^NTR^, and **(D)** TrkA /P75^NTR^ ratio. Mean values (±SE) obtained from three independent experiments (^**^P < 0.01 w.r.t. control; ^***^P < 0.001 w.r.t. control).

Figure 4 shows that NGF induced TrkA receptor internalization in absence of CuSO_4_ and ZnSO_4_; the presence of metal ions slightly inhibited this effect (Figures 4A,B).

Conversely, CuSO_4_ and ZnSO_4_ elicited the NGF(1–14)-induced internalization of TrkA. The acetylated peptide showed a weak receptor internalization not affected by the presence of CuSO_4_ and ZnSO_4_.

The P75^NTR^ internalization paralleled the TrkA pattern (Figures 4A,C). Similar to TrkA receptor internalization, NGF activity was more inhibited by CuSO_4_ and almost totally by ZnSO_4_.

The TrkA/ P75^NTR^ internalization ratio decreased in the presence of metal ions for NGF and NGF(1–14) but not for Ac-NGF(1–14) (Figure 4D).

### Effect of chelator bathocuproine disulfonic acid (BCS) on TrkA signaling induced by NGF(1–14) in the presence of CuSO_4_ and ZnSO_4_

**Figure 5:**
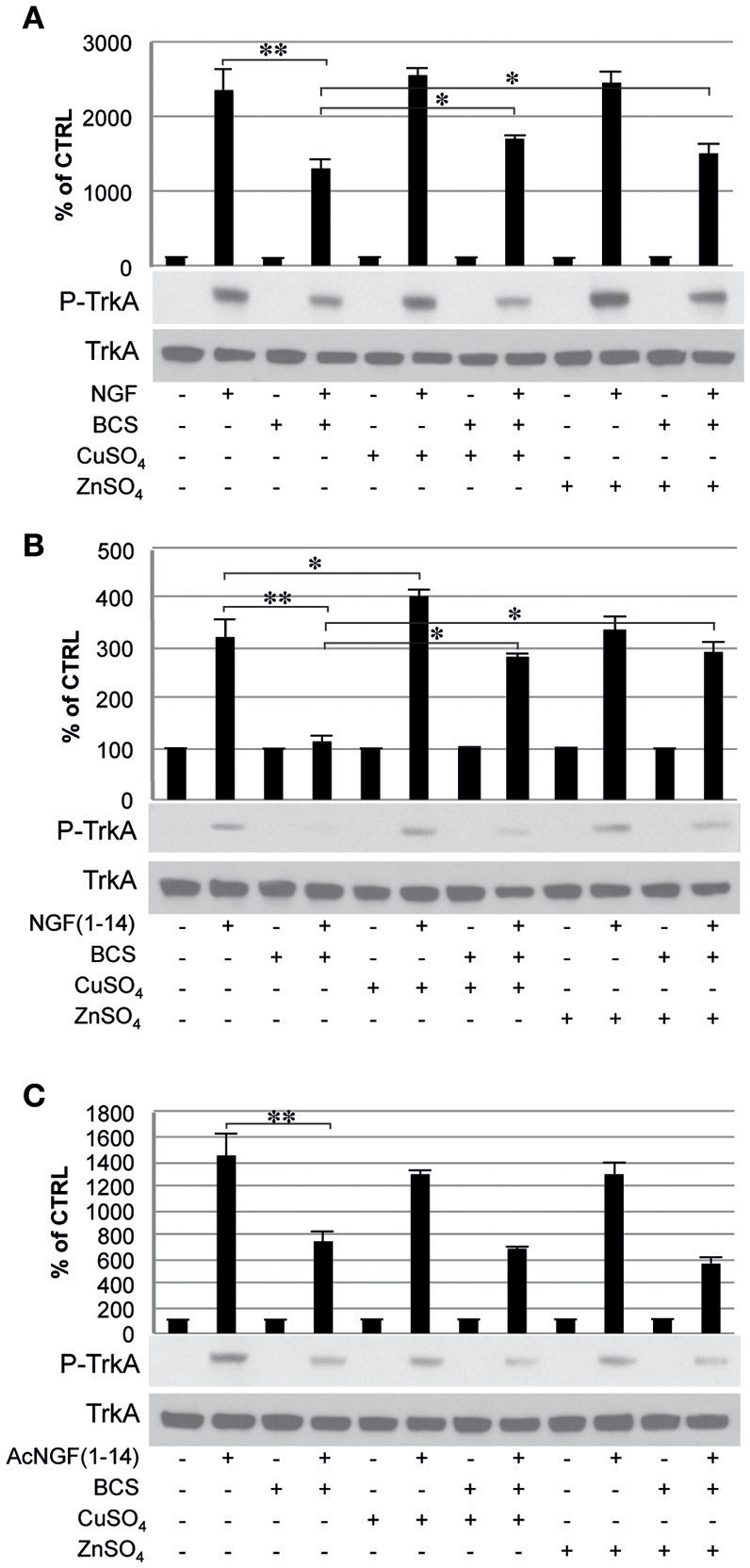
**Effect of bathocuproine disulfonic acid (BCS) on TrkA phosphorylation of PC12 cells**. PC12 cells un-pre-treated or pre-treated 24 h before with 50 μM BCS stimulated for 10 min in the presence or absence of Cu^2+^ and Zn^2+^with: **(A)** 50 ng/ml NGF, **(B)** 50 μM NGF(1–14), **(C)** 50 μM AcNGF(1–14). The top panels show the mean values (±SE) of three separate experiments, the bottom panels show a representative experiment of three (^*^P < 0.05 w.r.t. control; ^**^P < 0.01 w.r.t. control).

To evaluate whether the metals in the medium are essential in the TrkA signaling triggered by NGF and NGF(1–14) western blot analyses were performed in PC12 cells un-pre-treated or pre-treated 24 h before with BCS (a membrane-impermeable extracellular strong metal chelating agent), in the presence or absence of CuSO_4_ and ZnSO_4_ (Figure 5).

In cells incubated with NGF (50 ng/ml), Tyr-490 phosphorylation of TrkA was partially inhibited by pre-treatment with BCS (50 μM) (*P* < 0.01); this effect was partially restored by the presence of CuSO_4_ and ZnSO_4_ (*P* < 0.05) (Figure 5A).

In cells incubated with NGF(1–14) in the presence or in absence of CuSO_4_, Tyr-490 phosphorylation of TrkA, was significantly more potent (*P* < 0.05). The presence of BCS totally blocked the Tyr-490 phosphorylation of TrkA stimulated by NGF(1–14) (50 μM) (*P* < 0.01); this effect was partially inhibited by the presence of metal ions (*p* < 0.05) (Figure 5B).

**Figure 6:**
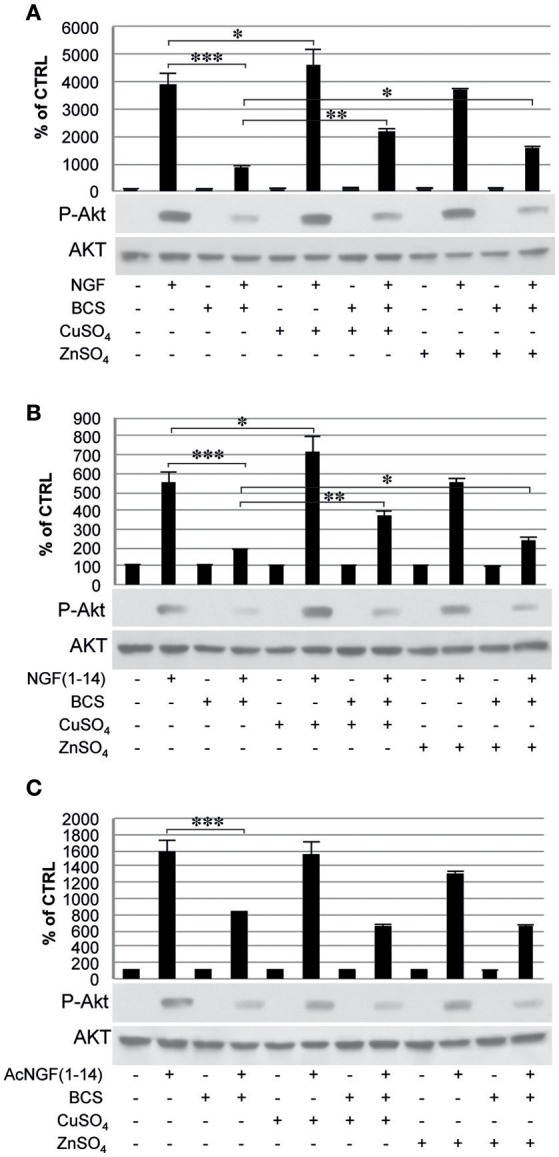
**Effect of bathocuproine disulfonic acid (BCS) on Akt phosphorylation of PC12 cells**. PC12 cells un-pre-treated or pre-treated 24 h before with 50 μM BCS stimulated for 10 min in the presence or absence of Cu^2+^ and Zn^2+^ with: **(A)** 50 ng/ml NGF, **(B)** 50 μM NGF(1–14), and **(C)** 50 μM Ac-NGF(1–14). The top panels show the mean values (±SE) of three separate experiments, the bottom panels show a representative experiment of three (^*^P < 0.05 w.r.t. control; ^**^P < 0.01 w.r.t. control; ^***^P < 0.001 w.r.t. control).

The Tyr-490 phosphorylation of TrkA stimulated by Ac-NGF(1–14) (50 μM) was inhibited by pre-treatment with BCS, but this effect was not restored by the presence of CuSO_4_ and ZnSO_4_ (Figure 5C). The AKT phosphorylation on Ser-473, in response to the stimulation with the ligands used, followed a pattern similar to that observed for TrkA phosphorylation, although ZnSO_4_ was less effective to restore the inhibitory effect of BCS (Figure 6). AKT phosphorylation on Ser-473 was significantly more activated in the presence of CuSO_4_ only when stimulated with NGF(1–14) (*P* < 0.05) (Figures 6A,B).

**Figure 7:**
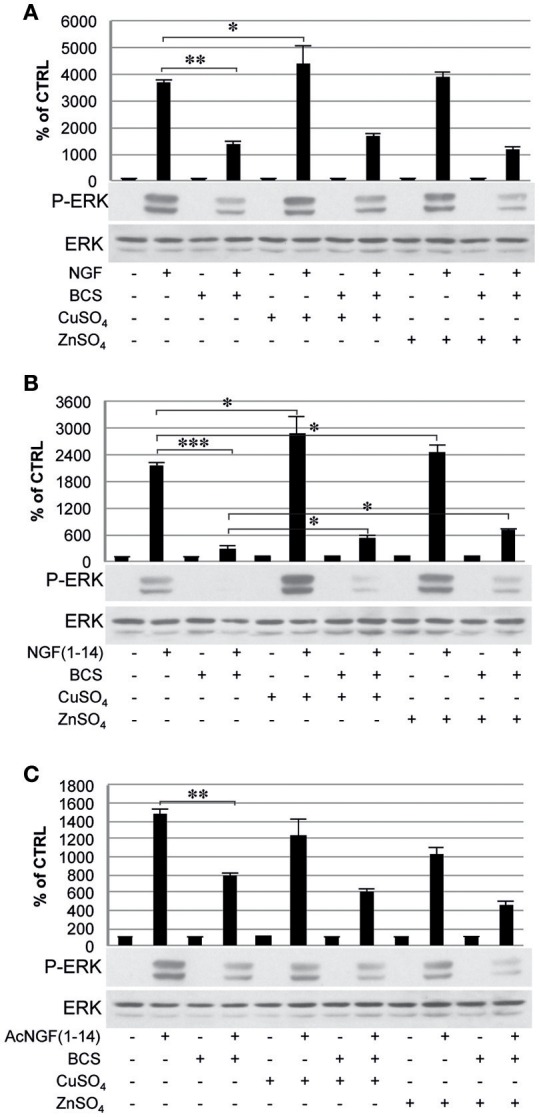
**Effect of bathocuproine disulfonic acid (BCS) on ERK 1/2 phosphorylation of PC12 cells**. PC12 cells un-pre-treated or pre-treated 24 h before with 50 μM BCS stimulated for 10 min in the presence or absence of Cu^2+^ and Zn^2+^ with: **(A)** 50 ng/ml NGF, **(B)** 50 μM NGF(1–14), and **(C)** 50 μM Ac-NGF(1–14). The top panels show the mean values (±SE) of three separate experiments, the bottom panels show a representative experiment of three (^*^P < 0.05 w.r.t. control; ^**^P < 0.01 w.r.t. control; ^***^P < 0.001 w.r.t. control).

The ERK 1/2 phosphorylation was also inhibited by pre-treatment with BCS (Figure 7), but not in the presence of metal ions when stimulated with NGF(1–14) (*P* < 0.05) (Figure 7B). On the other hands, ERK 1/2 phosphorylation was activated in the presence of CuSO_4_ when stimulated with either NGF or NGF(1–14) (*P* < 0.05) (Figures 7A,B) and in the presence of ZnSO_4_, only for cells stimulated with NGF(1–14) (*P* < 0.05) (Figure 7B).

It must be noted that in a previous work NGF(1–14) 100 μM found unable to induce ERK1/2 phosphorylation in PC12 cells (Travaglia et al., 2015). The different effect observed in the present work is explained in terms of the aging of the cell model used. Indeed, although PC12 cells were used in both cases, here new early passages (<20) PC12 cells were used, with an expected higher responsiveness to NGF (Kinarivala et al., 2016).

**Figure 8:**
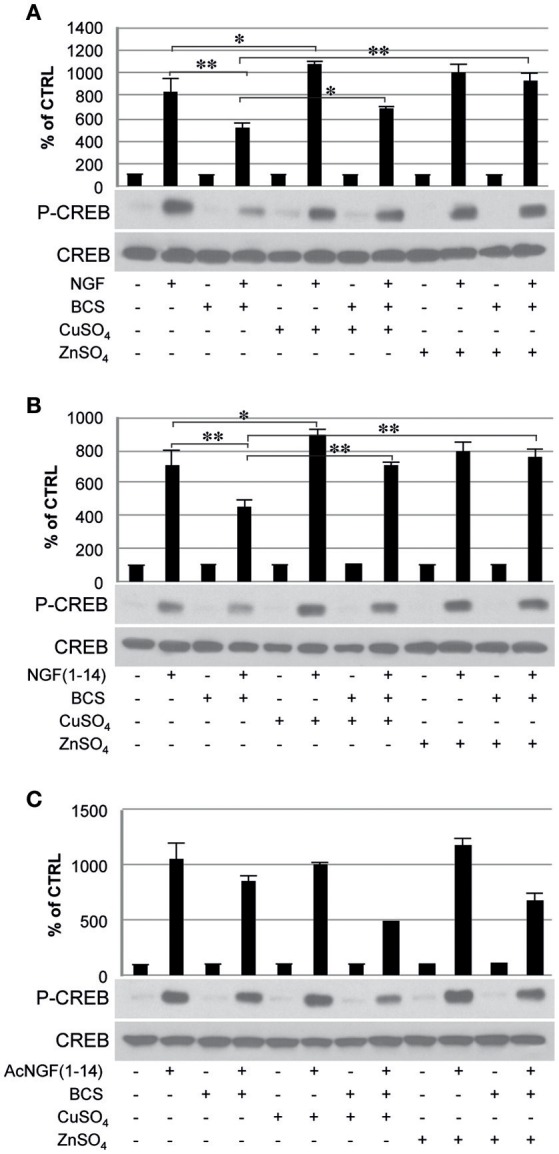
**Effect of bathocuproine disulfonic acid (BCS) on CREB phosphorylation of PC12 cells**. PC12 cells un-pre-treated or pre-treated 24 h before with 50 μM BCS stimulated for 10 min in the presence or absence of Cu^2+^ and Zn^2+^ with: **(A)** 50 ng/ml NGF, **(B)** 50 μM NGF(1–14), and **(C)** 50 μM Ac-NGF(1–14). The top panels show the mean values (±SE) of three separate experiments, the bottom panels show a representative experiment of three (^*^P < 0.05 w.r.t. control; ^**^P < 0.01 w.r.t. control).

The inhibitory effect of BCS on CREB phosphorylation at Ser 133 was evident in response to both NGF and NGF(1–14) (*P* < 0.01) (Figure 8). When stimulated with NGF or NGF(1–14), the Ser-133 phosphorylation of CREB increased markedly, in the presence of CuSO_4_ (Figures 8A,B). This effect less evident in cells stimulated with Ac-NGF(1–14) (Figure 8C).

### Effect of NGF(1–14) on BDNF mRNA expression of PC12 in the presence or absence of CuSO_4_ and ZnSO_4_

Brain-derived neurotrophic factor (BDNF) is a neurotrophin modulating survival, neurogenesis and differentiation of neuronal cells, the branching and survival of differentiated neurons, and the formation and maturation of the dendritic spine and synapses, influencing learning and memory.

To investigate the effect of NGF(1–14) on BDNF mRNA levels, in the presence or not of metal ions, PC12 cells were untreated or treated for 24 h with NGF (50 ng/ml), NGF(1–14) (10 μM), or Ac-NGF(1–14) (10 μM) in the presence or absence of CuSO_4_ or ZnSO_4_ (1 μM).

**Figure 9:**
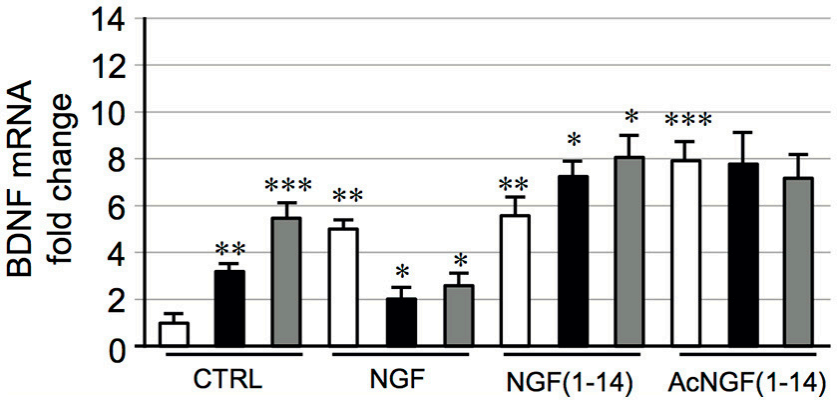
**Effect of NGF(1–14) on the expression of BDNF in PC12 cells**. PC12 cells incubated with NGF, NGF(1–14) and AcNGF(1–14) in absence (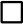) or in the presence of CuSO_4_ (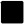) and ZnSO_4_ (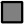) for 24 h. Levels of BDNF mRNA determined by real-time quantitative RT-PCR. Data normalized with respect to the expression level of B2M mRNA. Results are given as fold-chang of untreated cells. Data are the mean ± SEM of four independent experiments (^*^P < 0.05 w.r.t. control; ^**^P < 0.01 w.r.t. control; ^***^P < 0.001 w.r.t. control).

Figure 9 shows that CuSO_4_ and ZnSO_4_ a 3- and 5-fold increase of the BDNF mRNA levels respectively (*P* < 0.01 and *P* < 0.001). The NGF a 5-fold increase of BDNF mRNA levels compared to the untreated control (*P* < 0.01); this effect significantly in the presence of CuSO_4_ or ZnSO_4_ (*P* < 0.05).

Remarkably, NGF(1–14) and Ac-NGF(1–14) respectively a 5- and 8-fold increase of the BDNF mRNA levels (*P* < 0.01 and *P* < 0.001). At variance with NGF, for NGF(1–14) a significant increase of the BDNF mRNA levels in comparison to NGF(1–14) alone (*P* < 0.05) was found in the presence of CuSO_4_ or ZnSO_4_. The presence of metals not affect the increase of BDNF mRNA induced by Ac-NGF(1–14).

### Confocal microscopy analysis of the cellular uptake of fluorescein-labeled NGF(1–14) and Ac-NGF(1–14) track dynamic influx processes

Live cell confocal imaging in real-time of the cells upon the addition of N-terminal free amino peptide and its acetylated form was performed to visualize, at cellular and nuclear level, the peptides uptake by the PC12 cells. To this purpose, fluorescent NGF(1–14)FAM and Ac-NGF(1–14)FAM, with the carboxyfluorescein (FAM) moiety covalently bound to the peptide C-terminus, were used.

**Figure 10:**
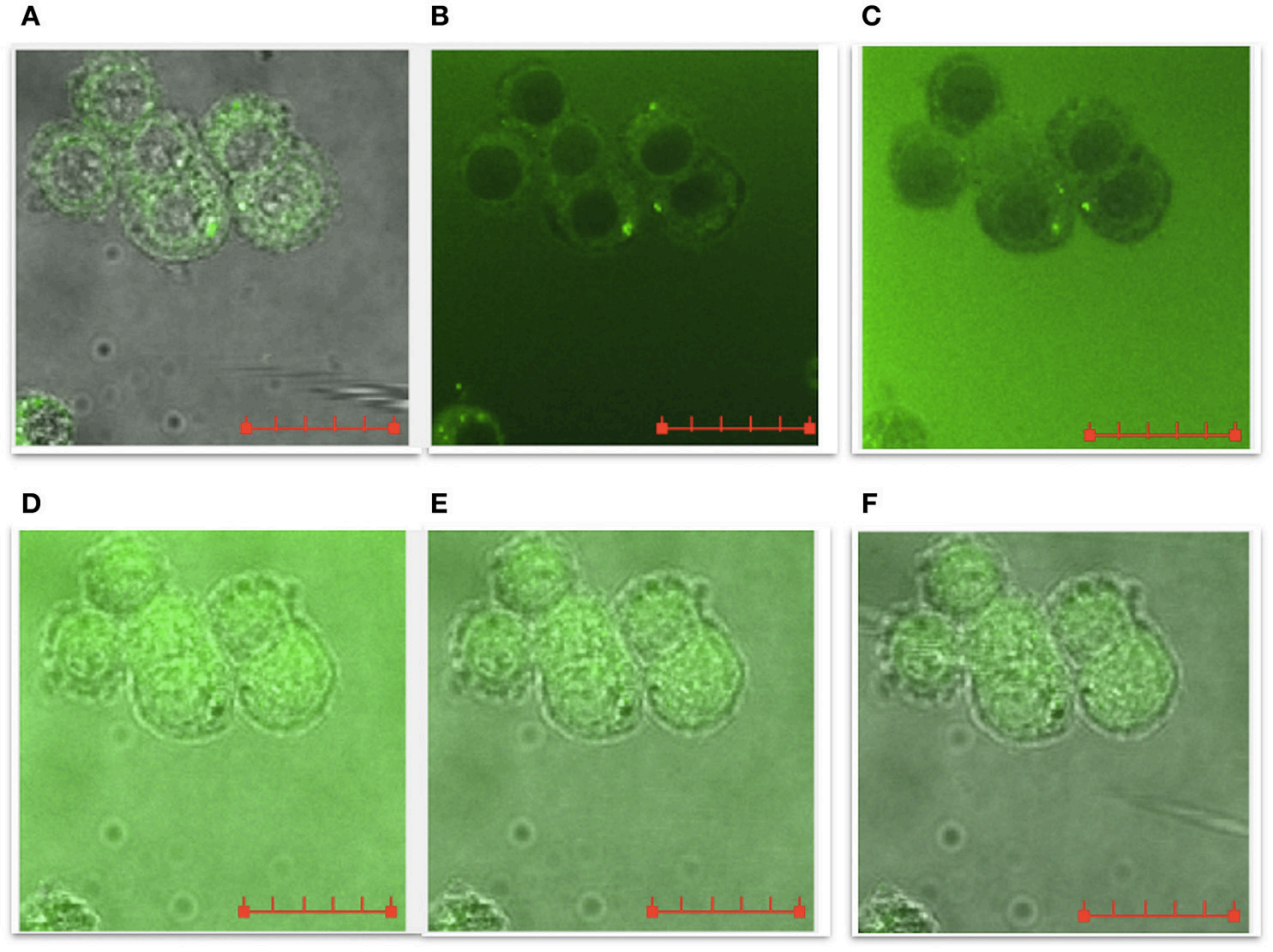
**LSM time-course merged confocal fluorescence (FAM emission recorded with excitation/emission wavelengths of 488/500–530 nm) and bright field optical images of PC12 cells treated with 10 μM NGF(1–14)FAM, added to the cell medium after an incubation time of: (A)** 0, **(B)** 1 min, **(C)** 4 min, **(D)** 7 min, **(E)** 8 min, **(F)** 10 min. The. Scale bar = 20 μm.

The representative time-course images showed in Figure 10 pointed out that: (i) the cellular uptake of the peptides was a quick process, occurring on the timescale of minutes at the used experimental conditions (i.e., 10 μM peptide at 37°C); (ii) the peptide molecules completely permeated the PC12 cells, including the nuclei; (iii) the process of cellular uptake was reversible, as the cells, turned-on by the green emission of the FAM-labeled peptide molecules, rapidly recovered to their original autofluorescence. The representative images for the whole set of samples are reported in Figure S1.

**Figure 11:**
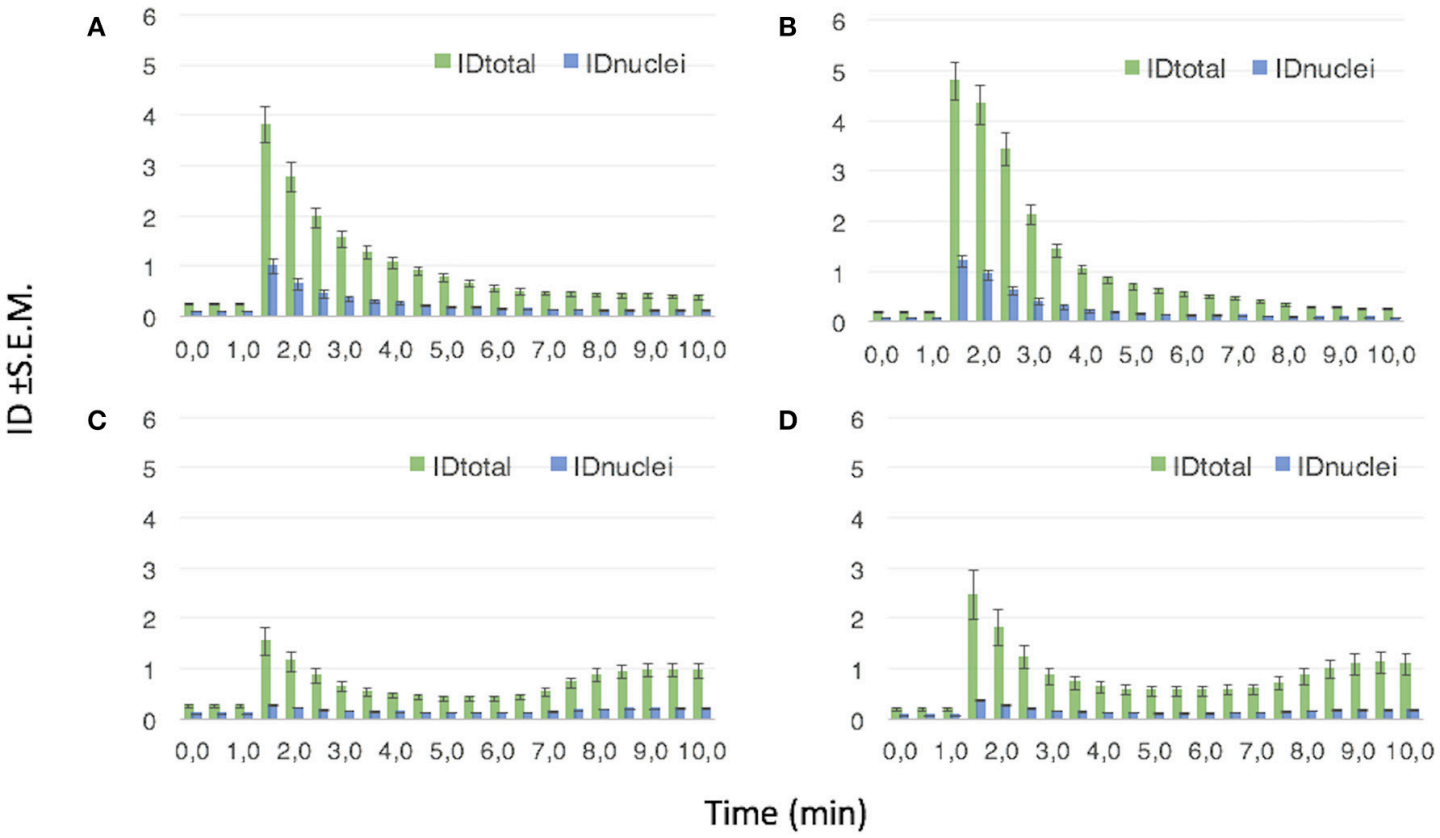
**Mean ID values ±S.E.M. from the analysis of ROIs corresponding to the whole cell (IDt_**otal**_) and the cell nuclei (ID_**nuclei**_), respectively**. The FAM emission (λex/em = 488/500–530 nm) is recorded in PC12 cells treated with: **(A)** 10 μM NGF(1–14)FAM; **(B)** 10 μM NGF(1–14)FAM:CuSO_4_ (1:1 molar ratio); **(C)** 10 μM Ac-NGF(1–14)FAM; **(D)** 10 μM Ac-NGF(1–14)FAM:CuSO_4_ (1:1 molar ratio).

Figure 11 reports the quantitative analysis of the FAM fluorescence (in terms of integrated density, ID, values) for NGF(1–14)FAM (Figure 11A), Ac-NGF(1–14)FAM (Figure 11B), NGF(1–14)FAM:Cu^+2^ (Figure 11C), and Ac-NGF(1–14)FAM:Cu^2+^ (Figure 11D).

The evident trend displayed that both NGF(1–14)FAM and Ac-NGF(1–14)FAM were internalized by the cells. The uptake of Ac-NGF(1–14)FAM (Figure 11C) was slightly lower than the cell entry measured for NGF(1–14)FAM (Figure 11A). However, whereas the latter exhibited an exponential ID decay, which can be interpreted as an efflux process of the peptide after the initial cellular uptake (in the time scale investigated and at the used experimental conditions), the acetylated peptide showed a sinusoidal curve trend, likely related to dynamic efflux/re-entry processes.

For both peptides, the two different trends in the measured IDs, i.e., exponential decay for NGF(1–14)FAM and sinusoidal for Ac-NGF(1–14)FAM, were maintained also when supplemented to cells together with copper ions.

In addition, the ratio of the fluorescence intensity measured in the nuclei with respect to that measured in the whole cell (ID_nuclei_/ID_total_), revealed another significant difference between the free-amino peptide and the acetylated one. Indeed, at the maximum of cell internalization (i.e., about 1.5 min on the x-axis in the plots of Figure 11), such a ratio was about 25% for NGF(1–14)FAM, both in the absence and in the presence of copper, about 18% for Ac-NGF(1–14)FAM and 15% for Ac-NGF(1–14)FAM:CuSO_4_. The gap observed for the acetylated peptide in the absence or presence of copper ions was still maintained and even increased in the minutes following the initial uptake, thus Ac-NGF(1–14)FAM exhibited a generally lower presence in the nuclei compared to the total peptide uptake.

### NGF(1–14) displays a copper-ionophore activity in PC12 cells

To evaluate the effect of NGF(1–14) and Ac-NGF(1–14) on the intracellular copper trafficking, confocal microscopy imaging was used to scrutinize the cells stained with the intracellular probe of monovalent copper **CS1** (Rampazzo et al., 2011).

**Figure 12:**
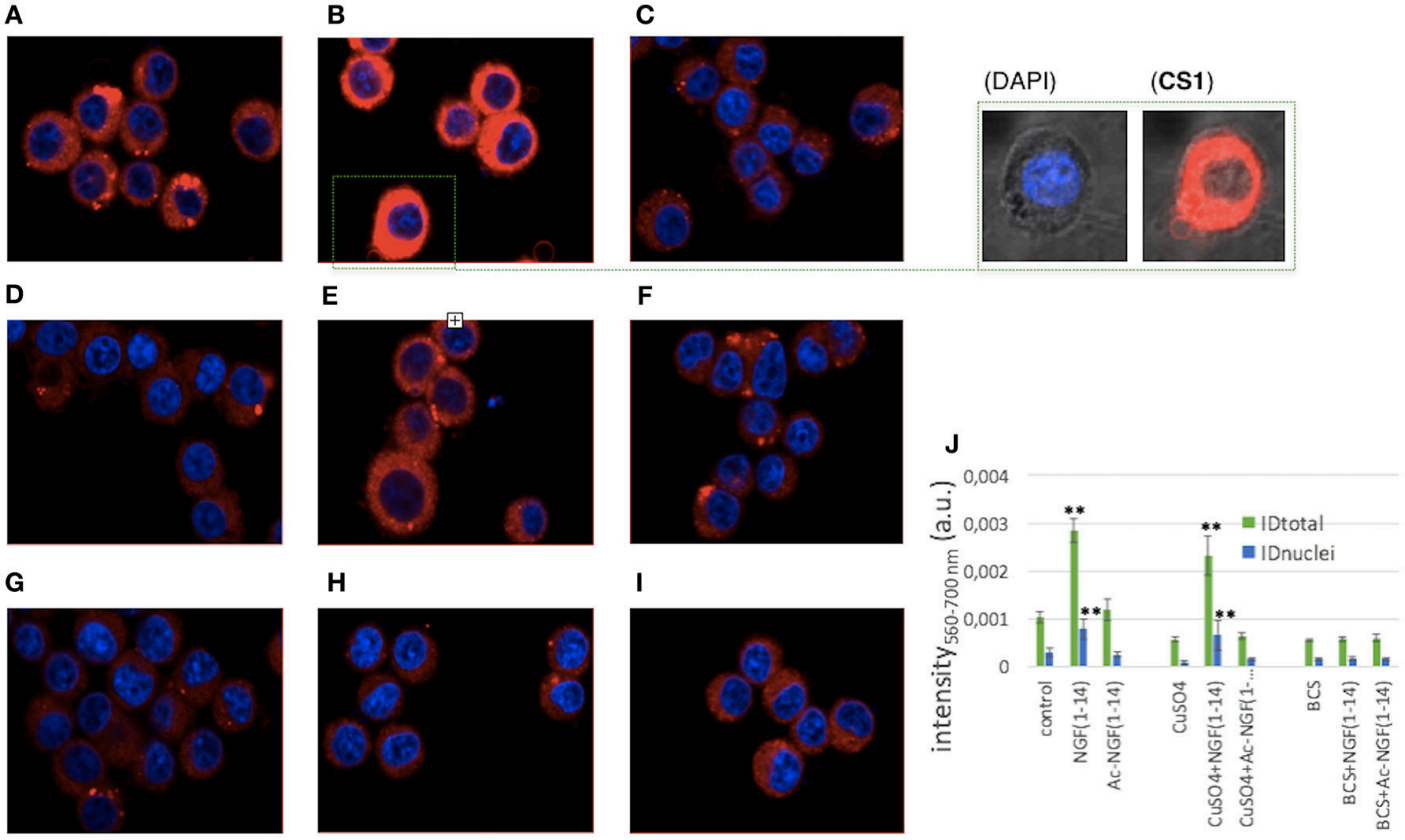
**Merged LSM fluorescence images of DAPI (nuclear staining, λex/em = 405/425–475 nm) and CS1 (monovalent intracellular copper reporter, λex/em = 488/500–530 nm) for PC12 cells after the different treatments. (A)** untreated; **(B)** 10 μM NGF(1–14)FAM (5 min incubation); **(C)** 10 μM Ac-NGF(1–14) (5 min incubation); **(D)** 100 μM CuSO_4_ (1 h incubation); **(E)** 100 μM CuSO_4_ (1 h pre-incubation) + 10 μM NGF(1–14)FAM (5 min incubation); **(F)** 100 μM CuSO_4_ (1 h pre-incubation) + 10 μM Ac-NGF(1–14)FAM (5 min incubation); **(G)** 50 μM BCS (3 h incubation), **(H)** 50 μM BCS (3 h pre-incubation) + 10 μM NGF(1–14)FAM (5 min incubation); **(I)** 50 μM BCS (3 h pre-incubation) + 10 μM Ac-NGF(1–14)FAM (5 min incubation). Scale bar = 20 μm. In **(J)** mean ID (±S.E.M.) values calculated for intensities measured on the ROIs corresponding to the whole cell (ID_total_) or to the nuclei (ID_nuclei_). (^**^P < 0.01 w.r.t. ID_total_ or ID_nuclei_ control).

The micrographs in Figure 12 show the response to the Cu^+^ reporter in a comparison of the PC12 cells for the different treatment conditions, i.e., free-amino vs. acetylated peptide, basal vs. copper-supplemented and copper-deprived medium.

The red emission of the fluorophore component (the BODIPY) of **CS1** was enhanced when the Cu^+^ chelator moiety (a thioether-rich receptor) binds to the intracellular monovalent copper (Miller et al., 2006). Figure 12 clearly demonstrates that **CS1** emission strongly increased for the cells treated with NGF(1–14) (Figure 12B) with respect to the control (Figure 12A), with a red fluorescence visible also in the nuclei. This observation is explained in terms of NGF(1–14) that acts as ionophore, i.e., able to bind extracellularly the Cu^2+^ ions in the basal medium and transport them at the cell membrane, where the redox conversion to the intracellular Cu^+^ occurs (Nevitt et al., 2012).

Since copper content in the basal media can reach concentrations up to micromolar (Huang et al., 2004), the experiments were repeated by supplementing the cell medium, before the peptide addition, with 100 μM CuSO_4_ for 1 h (Figures 12D–F) or with BCS pre-tretment for 3 h (Figures 12G–I).

As to cells pre-incubated with copper sulfate before of the peptide addition, only for NGF(1–14) (Figure 12E) a visible increase of fluorescence was detected, confirming its active role in the extracellular Cu^2+^ uptake/intracellular Cu^+^ mobilization machinery (Giuffrida et al., 2014).

For both CuSO_4_ (Figure 12D) and CuSO_4_+Ac-NGF(1–14) (Figure 12F) –treated cells, no significant increase of intracellular copper was observed in comparison to control. Such a result is not surprising owing to the used experimental conditions (i.e., the culture medium supplemented for 1 h with 100 μM CuSO_4_). Indeed, it is known that elevating the copper concentrations in the growth medium is able to enhance the cytoplasmic copper concentrations only after several hours of incubation time, depending on the cell line (Trusso Sfrazzetto et al., 2016).

The pre-incubation of the cells in the medium supplemented with BCS led to a relatively small but still noticeable decrease of the fluorescence intensity (Figure 12G) compared to the basal conditions. The addition of NGF(1–14) (Figure 12H) or Ac-NGF(1–14) (Figure 12I) did not result in any visible change of emission.

The quantitative analysis results (Figure 12J) evidenced statistically significant differences only for cells treated with NGF(1–14), which therefore is confirmed to play a role as ionophore, both in the basal and in the copper-supplemented medium. Noteworthy, such an effect of NGF(1–14)-induced increase of intracellular copper content is also significant in the nuclei.

## Discussion

### NGF-mimetic activity of NGF(1–4) and Ac-ANGF(1–14)

Peptide mimetics is raising a considerable interest owing to the versatility in the synthesis and the tunability of properties of small size peptides compared to the full-length protein (La Mendola et al., 2010; Forte et al., 2014). For example, a peptide can contact a few selected key regions of the target receptor rather than extensive protein surface (Skaper, 2011) allowing an easier design and evaluation of the functional binding activity.

In these regards, the N-terminal domain of NGF has been disclosed as essential for the stability of the NGF/TrkA complex (Berrera et al., 2006), where the formation of H-bonds at the protein−protein interface stabilized the functional NGF/TrkA-D5 binding (Settanni et al., 2003).

We recently reported that NGF(1–14), a linear peptide encompassing the 1–14 sequence of the human NGF protein, is able to activate the TrkA pathway, to induce the phosphorylation of CREB Ser-133, and to affect the PC12 proliferation rate (Travaglia et al., 2015). The NGF(1–14) peptide is the first example of linear peptide with NGF mimetic activity.

Another relevant finding on the NGF(1–14) peptide is that induced only minor morphological changes of PC12 cells, thus suggesting the selective activation of specific signaling pathways of the whole NGF protein (Travaglia et al., 2015).

It is known that the p75/TrKA receptors cooperation is necessary to transduce the entire pattern of NGF signaling. Therefore, to better understand whether the “selective” activation of TrkA receptor might drive/modulate specific NGF signaling, in the present study we performed a new series of experiments to elucidate on the NGF N-terminal peptide activity, in comparison with its acetylated form, and in the presence or not of metal ions. In particular, we carried out combined parallel tempering/docking simulations in order to assess the effects of the N-terminal acetylation of the NGF(1–14) peptide on its molecular recognition of domain 5 of TrkA (TrkA-D5).

These simulations were achieved through the structural data provided by the NGF/TrkA co-crystallization structure (Wiesmann et al., 1999). Noteworthy, we found that while the conformational states of Ac-NGF(1–14) were not affected by the acetylation, an abrupt alteration in the binding modes of Ac-NGF(1–14) to TrkA-D5 was instead observed. The most influenced residue was predicted as the N-terminal serine (S1), which is obviously highly exposed to the effects induced from the insertion of the acetyl group. In particular, S1 completely lost the contacts with histidine H343 or glutamic E295, both featuring the binding between NGF(1–14) and TrkA-D5. The forth histidine residue of Ac-NGF(1–14) H4, lost the contact with H291, which instead was present in NGF(1–14). However, the acetylated peptide, Ac-NGF(1–14), spans a larger number of non-covalent interactions with TrkA-D5 than the analog NGF(1–14), by the formation of hydrogen bonds or salt-bridge interactions.

It is to note that the NGF binding to TrkA, and the following induction of biochemical pathways, is mainly based on weak non-covalent interactions (Wiesmann et al., 1999). Therefore, the enhancing of such interactions can lead to an optimal peptidomimetic activity (Massa et al., 2003). Weak interactions have also been observed in other NGF-mimetic peptides, where docking simulations predicted the binding region of the peptide as a shallow hydrophobic cavity of TrkA (Scarpi et al., 2012). Furthermore, the presence of β-turn and loop structures have been related to an enhanced propensity for NGF mimicking activity, by considering the neurotrophin β-turn domains as the selective portions for the specific receptor binding (Brahimi et al., 2014). Intriguingly, the typical TrkA binding sites involve the NGF loop 2 (residues 40–49: GEVNINSVF), loop 4 (residues 91–97: TMDGKQA), the N terminus (residues 1–8: SSSHPIFH), and the C terminus (residues 111–115: VLSRK). Loops 1 and 4 based peptides (Xie and Longo, 2000; Massa et al., 2003) or a combination of them (Colangelo et al., 2008) have been rigorously studied and identified as NGF peptide mimetics in a number of contributions; the N-terminus domain, however, has been less investigated in the years.

### The effects of copper and zinc ions on the activity of NGF and NGF-mimic peptides in proliferation and differentiation, TrkA signaling, CREB phosphorylation and BDNF mRNA expression in PC12 cells

Metal ions, especially copper and zinc, are known to affect the NGF activity (Hwang et al., 2007; Travaglia et al., 2012b). Copper may be required for various aspects of NGF-stimulated neuronal differentiation. For example, copper has been found to promote neurotransmitter release from synaptosomes (Wang, 1999) and to facilitate neurite outgrowth.

Early clinical trials of Cu/Zn ionophores including clioquinol (CQ) and PBT2 have demonstrated promising outcomes in Alzheimer's disease (AD) patients (Lannfelt et al., 2008; Crouch et al., 2009; Faux et al., 2010). It has been generally observed that also the level of zinc transporters (ZnT3, ZIP 1, and 6 as well as ZnT1, 4, and 6) undergo various age-related changes in the brain, and in AD patients (Prakash et al., 2015). Indeed, treatment of ZnT3 KO mice with clioquinol has been found to restore the hippocampal zinc levels and up-regulated key proteins particularly relevant for cognition functions (e.g., presynaptic SNAP-25 and synaptophysin, post-synaptic spinophilin and PSD-95, cell supporting pro-BDNF and DCX, glutamate receptors AMPAR, and NMDAR2a/2b). CQ, being a chaperone able to trespass the BBB, rather than acting as metal chelator to remove metal ions, can redistribute zinc to brain post-synaptic targets leading to events such as TrkB activation and pro-BDNF conversion with beneficial effects on cognitive function in amyloid protein pre-cursor transgenic mice, AD model, and ZnT3 KO mice (White et al., 2006).

These effects may provide neuroprotective signaling changes in neurons, as also recently demonstrated by the observation of morphological changes, i.e., robust induction of neuritogenesis and neurite elongation, in PC12 neuronal-related cells after treatment by bis(thiosemicarbazonato)-copper complexes (Bica et al., 2014). Several new therapeutic strategies are currently aimed at regulating and restoring metal homeostasis (Opazo et al., 2014; Bharti et al., 2016). Interestingly, also four neurotrophic factor, namely BDNF, CNTF, PEDF, GDNF, have been reported to increase the intracellular Zn level in RPE (retinal pigmental epithelium) cells, modulating the expression of zinc transporters which increase zinc uptake (Leung et al., 2008).

In light of the above observations, NGF-mimicking peptides could both to re-establish a normal metal homeostasis and to potentiate the neurotrophic signaling. The experimental data presented here demonstrate significant differences between NGF and the NGF-mimetic peptides on PC12 proliferation/differentiation, in particular in the presence of copper and zinc ions (Figure 3). These findings open to speculations and to further investigations on the early events that occur upon receptor binding (Chao et al., 2006; Matusica and Coulson, 2014).

Here we confirm the NGF ability to induce TrkA and p75NTR receptors internalization, and demonstrate that NGF(1–14) and Ac-NGF(1–14) imitate such an effect (Figure 4). Moreover, different activity was observed in the presence of metals ions: the NGF-induced receptors internalization was inhibited, whereas that of NGF(1–14) ameliorated, and no significant differences in the presence or absence of metals were observed for Ac-NGF(1–14).

The absence of metals effects on the Ac-NGF(1–14)-induced receptors internalization supports the hypothesis of a direct effect of metals on peptides conformation although a metal-related change in the endosome machinery cannot be excluded.

The typical TrkA engagement by NGF (Biarc et al., 2013) was also reproduced using the NGF-mimetic peptides NGF(1–14) and AcNGF(1–14), which were able to phosphorylate Tyr-490, thus triggering an intracellular signaling with strength similar to that elicited after NGF stimulation (Figure 5). This, in turn, was followed by Akt phosphorylation (Ser-473) (Figure 6), and the downstream ERK1/2 phosphorylation that paralleled the pattern observed for Akt phosphorylation (Figure 7).

The Ras-MAPK/Erk signaling cascade is essential for neurotrophin-promoted differentiation of neuronal cells, and transient vs. prolonged MAPK-activation might also be associated with mitogenic- or proliferation-promoting responses (Katz et al., 2007). The ERK1/2 are MAPKs activated by TrkA and are known to phosphorylate and activate the downstream transcription factor CREB (as well as Elk-1, and MEF2), to regulate target gene expression and to contribute to neuronal differentiation and survival (Riccio et al., 1999; Pearson et al., 2001).

Indeed, by the investigation of CREB phosphorylation on Ser-133, we observed that the stimulation of TrkA by NGF and NGF-mimetic peptides was able to activate the canonical end of the cascade (Figure 8). Such a finding demonstrates that our NGF-mimetic peptides can reproduce the signal transduction of the whole protein, being therefore good candidates for further pre-clinical studies.

Many evidences suggest that metal ions could play a role in the NGF and NGF(1–14) signal transduction triggering (Travaglia et al., 2011). Here we demonstrate that copper addition stimulates the NGF(1–14)-mediated TrkA phosphorylation (Figure 5), whereas both the NGF and NGF(1–14) induce Akt, ERK and CREB phosphorylation (Figures 6–8).

In addition, the crucial role of the metal is evidenced also by a general inhibitory effect on both NGF and related peptides in the signaling cascade in the presence of BCS (Figures 5–8). Indeed, at the same experimental condition of metal deprivation, the recovery of NTs-related activities is achieved by copper supplementation to NGF and NGF(1–14) but, as expected, is ineffective for Ac-NGF(1–14) (Figures 5–8). The functional recovery found upon zinc addition, again except than for Ac-NGF(1–14)-mediated signaling, clearly demonstrates the critical role also of this metal (Figures 5–8).

It is to note that NGF(1–14) binds to Zn^2+^ with a higher stability constant than Ac-NGF(1–14), similarly to the trend observed for the binding affinities toward Cu^2+^ of the two peptides (Travaglia et al., 2011). This might explain the higher ionophore activity showed by NGF(1–14) in comparison to its acetylated analogous (Figures 10–12).

The metals homeostatic machinery could be affected or involved in the NGF route to signaling, and the strong disturbance of BCS pre-treatment can affect cell physiology and specific signaling knots. The issue has never been explored and therefore represents a new challenge in the study of neurotrophins activities, as well as memory formation. The restorative effect of NGF treatment is known to be related to its cell internalization and to CREB phosphorylation, followed by CREB-induced gene expression. These peptides are the first linear N-terminal NGF sequences with a direct role in the activation of the Akt-CREB signaling cascade and therefore with high potentials for further pre-clinical approaches. Noteworthy, nanomedicine approaches, e.g., by the immobilization of these peptides at the surface of proper nanoparticle platform, can help in the actual translation of these systems from the bench to the bedside (Di Pietro et al., 2016).

The results in this new study show the ability of NGF(1–14) and its acetylated derivative to up-regulate BDNF mRNA, in PC12 cells and further demonstrate the involvement of copper and zinc ions in these processes. Indeed, the treatment with CuSO_4_ or ZnSO_4_, induces an increase respectively of 3-, or 5-fold) of the BDNF mRNA levels in comparison to the untreated control, that is comparable to that elicited by NGF treatment (5-fold increase) (Figure 9). Interestingly, while the co-treatment with CuSO_4_ or ZnSO_4_ significantly down-regulate BDNF mRNA for NGF, a 8-fold increase of the BDNF mRNA levels is found for Ac-NGF(1–14) (Figure 9).

These results appear to be irrespective of the effects on the upstream kinases, but it is possible to argue that different routes, here not explored, could be responsible of such important outcome. In any case, the BDNF mRNA expression parallels the CREB phosphorylation. The relevance of BDNF in the process of synaptic plasticity and memory formation, as well as in neuroprotective responses, makes our peptides suitable for a large plethora of hypothetical approaches. At the same time, the metal ion involvement here exposed puts in evidence the importance of a fundamental bioinorganic perspective.

### CREB phosphorylation and BDNF mRNA expression by NGF(1–14) might correlate with its copper-ionophore activity

In correlation to the observed effects on the CREB activation and the increase of mRNA levels of BDNF, the confocal microscopy results of a reversible cellular uptake, especially in the nuclei, for both NGF(1–14) and Ac-NGF(1–14) peptides. This is relevant to confirm the role of the selected sequence (1–14) of NGF to mimic the whole protein. In particular, the LSM studies demonstrate that both NGF(1–14) and its acetylated derivative Ac-NGF(1–14) can be internalized by PC12 cells in a fast process (time scale of seconds-minutes) of cellular influx and efflux. The main difference observed between the two peptides is a more dynamic uptake process for Ac-NGF(1–14), which results in a re-entry effect of the peptide, not observed for NGF(1–14).

A second relevant LSM result is the role of copper ions in the modulation of peptide sub-cellular localization. In particular, Ac-NGF(1–14) that binds copper with a lower stability constant and a different coordination environment than NGF(1–14) (Travaglia et al., 2011), exhibits a nuclear localization with respect to the total cellular uptake (quantified by the ID_nuclei_/ID_total_ ratio) significantly lower in the presence of copper ions. On the contrary, the ID_nuclei_/ID_total_ ratio in the cellular uptake of NGF(1–14) is comparable both without and with the addition of copper ions.

Thirdly, LSM measurements demonstrate the crucial role in the copper trafficking played by the free amino group at the N-terminal residue. Indeed, only NGF(1–14)-treated cells increase their intracellular content of monovalent copper, also in the nuclei. This results hold both in the basal (typically containing Cu^2+^ ions at concentrations up to the micromolar range) and in the copper-supplemented medium. According to the reversible cellular uptake/efflux process described above, an actual role of NGF(1–4) as ionophore is therefore demonstrated.

As known, metallostasis (Milardi and Rizzarelli, 2011) is a complex and dynamic machinery in the spatio-temporal scale (La Mendola et al., 2016; Trusso Sfrazzetto et al., 2016). The used approach to track the intracellular copper needs a careful analysis of results for the correct data interpretation. Indeed, the fluorescent synthetic probe **CS1**, selective and specific toward Cu^+^ ions, binds to the intracellular monovalent copper in competition with the other copper ligands. The comparison between the two peptides NGF(1–14) and Ac-NGF(1–14), with the unique difference of the presence of the free amino group, strongly supports the more relevant ionophore activity of NGF(1–14), thus providing a smart design route to achieve a modulation of copper homeostasis.

## Concluding remarks

In the present work predictive results of computational studies and experimental investigations on the NGF N-terminus peptide, NGF(1–14) and its acetylated form, Ac-NGF(1–14), were addressed to reinforce previous evidences (Travaglia et al., 2015) on the ability of this neurotrophin domain to mimic some biological features of the whole protein.

Non-covalent forces (hydrogen bonds, electrostatic and hydrophobic interactions) were found to assist the molecular recognition of the receptor TrkA by the two peptides, even if with slight differences. Such a recognition process leads to the phosphorylation of TrkA and the activation of some of its downstream targets. Experimental findings demonstrated that NGF(1–14) and Ac-NGF(1–14) induce the phosphorylation of Akt and ERK1/2, thus activating the related signaling pathways, particularly relevant for neuronal survival.

The pro-survival effect of NGF treatment is known to be due to its signaling, cell internalization, and CREB phosphorylation (Finkbeiner et al., 1997) followed by CREB-induced gene expression. Our findings indicate that both NGF(1–14) and Ac-NGF(1–14) activate the NGF signaling cascade and induce CREB phosphorylation. Noteworthy, CREB is a major transcriptional mediator of neuronal responses to neurotrophins and a key regulator in development and adaptive responses. CREB is involved in stimulus-dependent transcription, which mediates neuronal plasticity and axonal regeneration (Teng and Tang, 2006), memory consolidation (Lonze et al., 2002; Alberini, 2009; Kim et al., 2013; Bisaz et al., 2014) as well as metabolism (Leone et al., 2011).

**Scheme 2:**
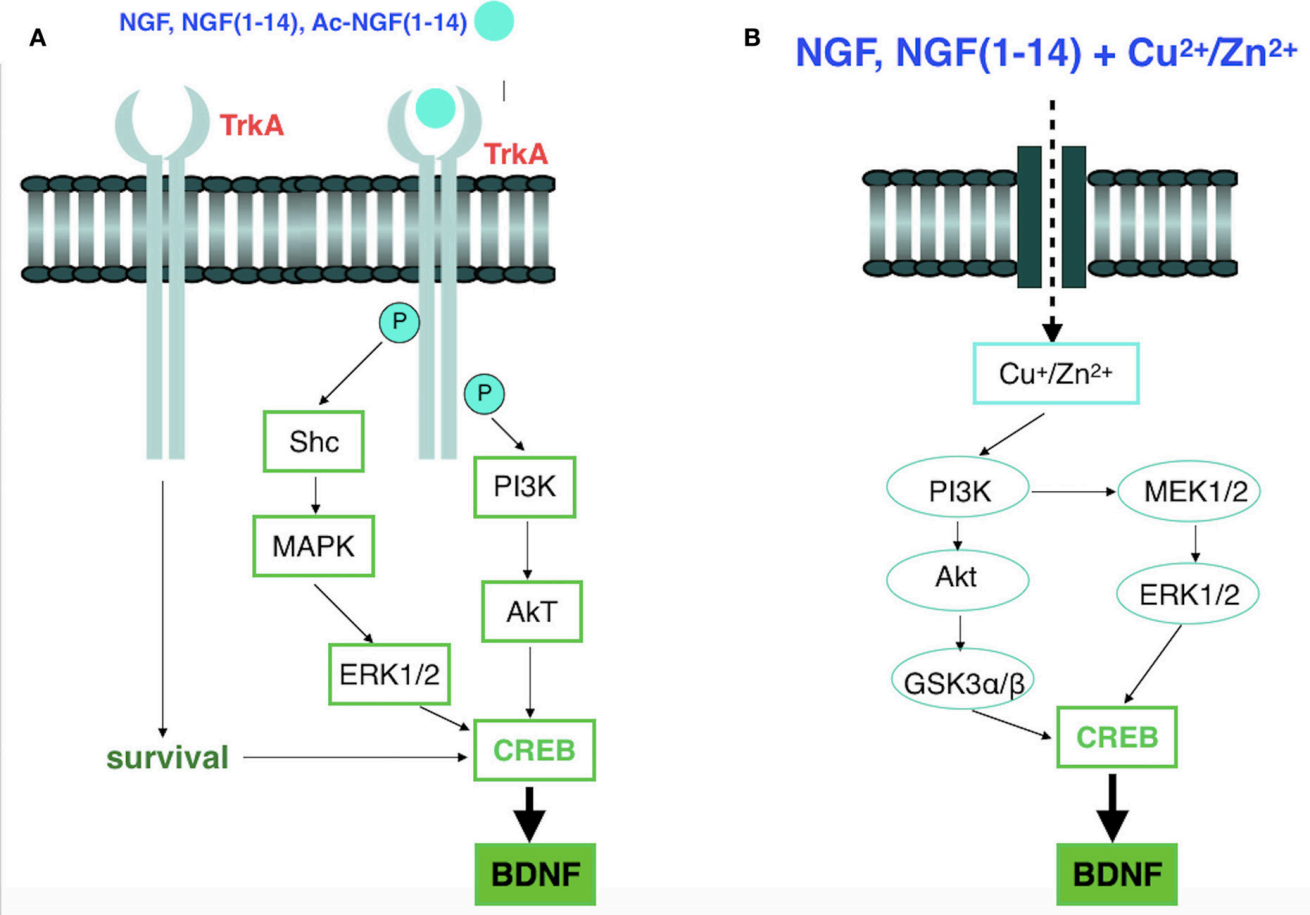
**(A)** Signaling of NGF receptor TrkA. The binding of NGF or NGF(1–14) or Ac-NGF(1–14) to TrkA causes its phosphorylation. Multiple signaling pathways, such as the PI3K/Akt, Ras/Raf/MEK/ERK1/2, or PLCγ/PKC, are activated, which eventually leads to different biological functions, including CREB phosphorylation and the expression of BDNF. **(B)** Schematic of copper and zinc influx, in the presence of NGF or NGF(1–14) and the following signaling pathways. NGF(1–14)-copper complexes enter the cell by an unknown process and activate signaling pathways involving PI3K activation. PI3K activates MEK1/2, resulting in phosphorylation of ERK1/2. PI3K also activates Akt via phosphorylation, which, in turn, mediates phosphorylation of GSK3. Upon phosphorylation, GSK3 and ERK1/2 potentiate activation of CREB and BDNF.

These findings demonstrate that NGF(1–14) and Ac-NGF(1–14) are the first monomer and linear peptides able to activate the NGF signaling cascade. In fact, as NGF, the two peptides induce CREB phosphorylation (Alberini, 2009) and BDNF gene expression (Altar et al., 1993) (Scheme 2).

Moreover, confocal microscopy results pointed to a ionophoric ability of NGF(1–14) and Ac-NGF(1–14), which favors the copper and zinc influx in a way that is correlated to the different metal affinity of the two peptides. Noteworthy, the metal ions in the presence of NGF(1–14) affect the Akt and ERK1/2 signaling pathway in a parallel way to that of the peptide alone; also, copper and zinc addition to NGF(1–14) increase CREB and BDNF expression. The influence of metal ions on the kinase cascade recalls the analogous behavior of clioquinol and other ionophores.

Such ionophores induce the degradation of the amyloid β-peptide (Aβ) by the metal-dependent signaling activation of PI3k and MAPK and the up-regulation of metalloprotease (MMP) activity (White et al., 2006; Crouch et al., 2009), respectively. The Scheme 2B shows the common metal-assisted kinase pathways that differ only in the final step, i.e., the production of MMP necessary to avoid the Aβ toxic oligomers (for clioquinol) respect to CREB activation and BDNF expression (for NGF(1–14) and NGF).

Studies on neuronal signal transduction associated with the memory formation (Sindreu and Storm, 2011) indicate that zinc can also inhibit tyrosine phosphatases by binding to their conserved catalytic domain (Brautigan et al., 1981; Haase and Maret, 2003; Redman et al., 2009). Namely, the inhibition of MAPK phosphatases by zinc is in agreement with previous observations in neuron cultures (Ho et al., 2008). However, how the synaptic zinc regulates selective signaling pathways via phosphatase inhibition remains an intriguing question.

**Scheme 3:**
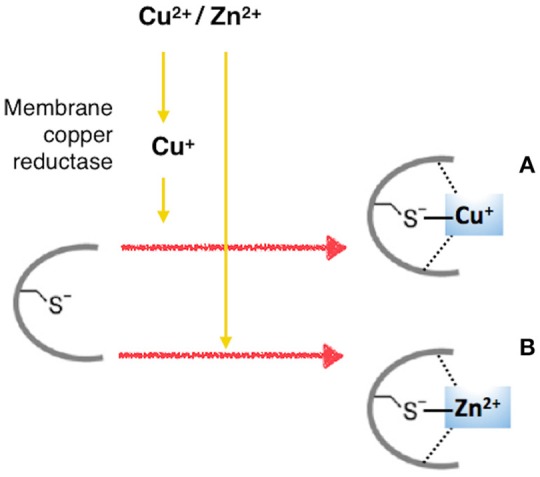
**Interaction of copper and zinc ions with protein tyrosine phosphatases (PTPases). (A)** Intracellularly, Cu^2+^ is reduced to Cu^+^, which interacts with the cysteine thiolate active site of PTPases. **(B)** Zn^2+^ inhibits PTPases, likely by the interaction with cysteine thiolate active sites.

The insulin-mimetic ability of Cu^2+^ and Zn^2+^ to activate the PI3K/Akt pathway by the interaction with insulin-receptor (IR) and IGF1-receptor (IGF1R) in HepG2 human hepatoma cells (Walter et al., 2006), which result in the phosphorylation and nuclear exclusion of transcription factor FoxO1a, has been recently questioned (Hamann et al., 2014). In this work copper affected overall tyrosine phosphorylation and, more specifically, tyrosine phosphorylation involved in Akt/FoxO signaling, by the inactivation of phosphatases [PTPase(s)], according to previous findings (Kim et al., 2000; Barthel et al., 2007). Such an inhibition of a PTPase might explain the broad spectrum of proteins whose tyrosine phosphorylation appears to be stimulated in the presence of copper. PTPases, all sharing the active site cysteine thiolate, would allow for an inhibition not only by copper but also by zinc (Scheme 3).

Zn^2+^ inhibits PTEN, a PTPase-family phosphatase that is crucial to IR/Akt/FoxO signaling, but that acts downstream of IR/IGF1R, by dephosphorylating phosphatidylinositol-3′,4′,5′-trisphosphate, being required for a Zn-induced modulation of Akt phosphorylation (Plum et al., 2014). Obviously, the induction of phosphorylation by inhibition of dephosphorylation requires at least basal tyrosine kinase activity.

Bearing in mind these findings and our results, we can speculate that copper and zinc ions imitate the signaling effect of NGF, but they do not mimic the NGF mode of action.

## Author contributions

AT and DL synthesized the peptides, analyzed the data and wrote the paper; GP, CS, and AP performed the experiments, analyzed the data, wrote the paper; FG performed the experiments; VN analyzed the data and wrote the paper; ER conceived and designed the experiments, analyzed the data, wrote the paper.

### Conflict of interest statement

The authors declare that the research was conducted in the absence of any commercial or financial relationships that could be construed as a potential conflict of interest.

## Supplementary material

Figure S1 **Time-course merged confocal fluorescence and optical images of cells treated with: 10 μM NGF(1–14)FAM (A)**, 10 μM NGF(1–14)FAM:Cu **(B)**, 10 μM Ac-NGF(1–14)FAM, **(C)** and 10 μM Ac-NGF(1–14)FAM:Cu **(D)**.
