# Supplementary figures and images for: The Inorganic Side of NGF: Copper(II) and Zinc(II) Affect the NGF Mimicking Signaling of the N-Terminus Peptides Encompassing the Recognition Domain of TrkA Receptor
Source: Front Neurosci. 2016 Dec 20;10:569. doi: 10.3389/fnins.2016.00569 (PMC5201159; doi:10.3389/fnins.2016.00569)

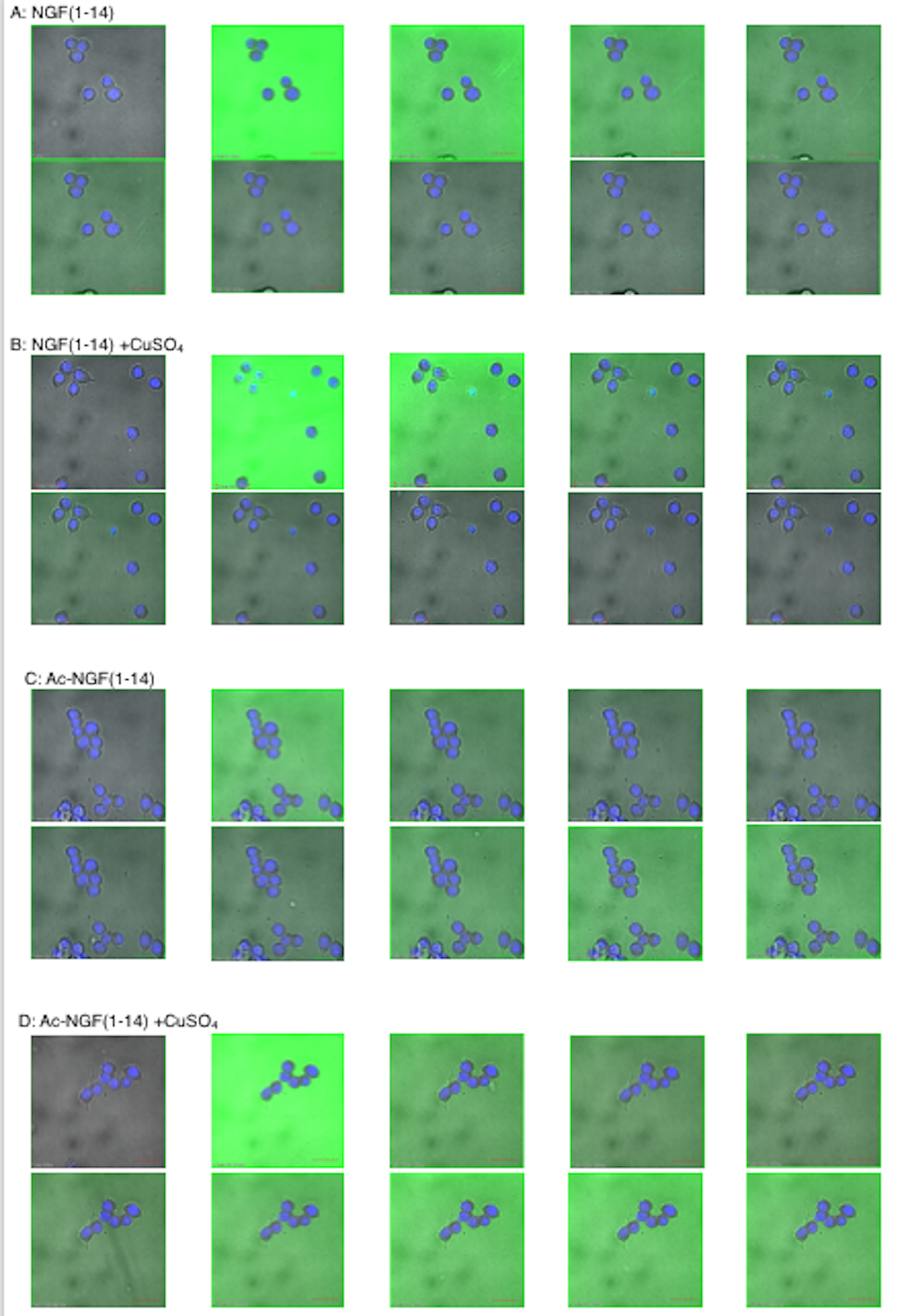

Supplement: Figure S1 — Time-course merged confocal fluorescence and optical images of cells treated with: 10 μM NGF(1–14)FAM (A), 10 μM NGF(1–14)FAM:Cu (B), 10 μM Ac-NGF(1–14)FAM, (C) and 10 μM Ac-NGF(1–14)FAM:Cu (D). [file Image1.TIFF]
